# Peptide receptor radionuclide therapy with somatostatin analogs beyond gastroenteropancreatic neuroendocrine tumors

**DOI:** 10.1111/jne.70013

**Published:** 2025-03-10

**Authors:** Giulia Santo, Gianpaolo di Santo, Francesco Cicone, Irene Virgolini

**Affiliations:** ^1^ Department of Nuclear Medicine Medical University of Innsbruck Innsbruck Austria; ^2^ Department of Experimental and Clinical Medicine “Magna Graecia” University of Catanzaro Catanzaro Italy

**Keywords:** neuroendocrine tumor, peptide receptor radionuclide therapy, SSTR

## Abstract

First isolated by Brazeau et al. in 1972, somatostatin (SST) is a neuropeptide known for regulating various signaling pathways through its specific cell surface receptors. Somatostatin receptors (SSTRs) comprise a family of five G protein‐coupled receptors that are widely distributed across the human body and are expressed by various tumor types. The growing understanding of their clinical potential led to the introduction of both cold and radiolabeled somatostatin analogs (SSAs), which have revolutionized the management of several cancers, especially neuroendocrine tumors. As a direct consequence, advances in peptide receptor radionuclide therapy (PRRT) over the last 30 years led to the approval of ^177^Lu‐DOTATATE for the treatment of gastroenteropancreatic neuroendocrine tumors (GEPNETs). Theoretically, any cancer patients whose tumors express SSTR, as demonstrated in vivo through SSTR‐based molecular imaging, could be candidates for PRRT, especially those with limited treatment options. However, evidence on the efficacy of PRRT in non‐GEPNET SSTR‐expressing tumors is limited, and mainly derived from small retrospective studies. Given the limited therapeutic options for advanced/metastatic patients, there is a clear need for randomized trials to formally approve PRRT with SSAs for patients who may benefit from this treatment, particularly in certain types of neuroendocrine neoplasms such as lung carcinoids, paragangliomas, and meningiomas, where high rates of disease control (up to 80%) can be achieved. In addition, emerging evidence supports the potential of combination therapies, alpha emitters, and non‐SSTR‐based radionuclide therapy in tumors beyond GEPNET. This review aims to provide a comprehensive overview of PRRT's role in cancers beyond GEPNET, exploring new possibilities and future directions for most SSTR highly expressing tumors.

## Graphical abstract

The thera(g)nostic approach with somatostatin analog‐based agents extends beyond gastroenteropancreatic neuroendocrine tumors. Understanding tumor biological mechanisms is essential for optimizing treatment strategies.

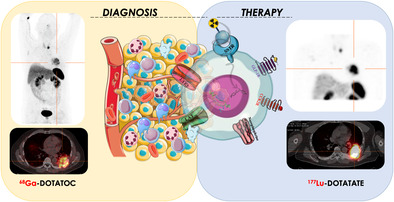

## 1 INTRODUCTION

Peptide receptor radionuclide therapy (PRRT) is a molecular radionuclide treatment involving the systemic administration of a radiolabeled peptide designed to target with high affinity and specificity cellular proteins, commonly cell surface receptors, such as somatostatin receptor (SSTR).^1^ Somatostatin (SST), a small cyclic neuropeptide first isolated by Brazeau et al. in 1972,^2^ is known for regulating a wide range of specific targets in the endocrine, gastrointestinal, and nervous systems, where it inhibits the release of hormones, growth factors, and cytokines.^3^ SST exerts its effects through five subtypes of cell surface receptors (SSTR1, SSTR2A and SSTR2B, SSTR3, SSTR4, and SSTR5), which belong to the G protein‐coupled receptor family.^4^ These receptors are widely distributed throughout the human body and are expressed by various tumors,^5^ particularly those derived from the neural crest.

The development of radiolabeled somatostatin analogs (SSAs) has significantly transformed both the diagnostic and therapeutic management of SSTR‐expressing tumors, particularly neuroendocrine neoplasms (NENs), a heterogeneous group of tumors arising from cells of neuroendocrine (NE) origin in many different organs. The landmark NETTER‐1 trial^6^ has led to the formal approval of ^177^Lu‐DOTATATE (Lutathera; Advanced Accelerator Applications USA, Inc., Millburn, NJ) by both the European Medicines Agency (EMA) and the US Food and Drug Administration (FDA) as a second‐line treatment in patients who progressed after cold SSAs. However, this study included only patients with advanced gastroenteropancreatic neuroendocrine tumors (GEPNETs) that account for only 30% of all NENs. In principle, all cancer patients with a tumor known to express high levels of SSTRs are eligible for high‐dose PRRT, especially those with limited or no alternative treatment options. Though, even if more than 20 years have passed since the Multicenter Analysis of a Universal Receptor Imaging and Treatment Initiative (MAURITIUS trial),^7^ evidence for the use of PRRT in NENs of other origins (non‐GEPNET) remains limited, primarily based on retrospective analyses involving small sample sizes. Understanding the biological mechanisms underlying GEPNET and non‐GEPNET tumors is essential for optimizing treatment strategies. Differences in frequently mutated genes have been observed between these tumor types, contributing to the identification of several molecular markers for predicting PRRT response, such as specific mutations, DNA methylation patterns, chromosomal abnormalities, and transcriptional alterations.^8^, ^9^ Furthermore, tumor immunogenicity and microenvironment are key determinants of response to therapy. Significant intratumoral heterogeneity has been documented in NENs, characterized by diverse immune cell populations, including conventional T cells, CD8+ T cells, NK cells, B cells, and plasma cells.^10^ Additionally, increased expression of IFNγ‐associated genes and heightened intratumoral T‐cell infiltration have been correlated with greater tumor aggressiveness.^11^ In the context of PRRT, a key factor influencing therapeutic efficacy is the heterogeneity of SSTR expression across various tumor types. Moreover, resistance mechanisms, such as receptor downregulation and tumor dedifferentiation, may develop differently across tumor entities, further impacting treatment outcomes.^12^ From a radiobiological perspective, additional factors, including the absorbed radiation dose, radiation type, and intrinsic tumor radiosensitivity, must also be carefully considered when evaluating different cancer types.^9^, ^13^

The aim of the present review was to summarize the evidence on the application of PRRT using SSAs in tumors beyond GEPNETs. In addition, future perspectives and new treatment approaches were also discussed.

### 1.1 Imaging of neuroendocrine tumors

The incidence of neuroendocrine tumors (NETs) has been rising over the past 30 years,^1^ largely due to advancements in diagnostic tools such as specific immunohistochemical methods and the progress of molecular imaging. Historically, scintigraphy with radiolabeled SSAs, first with ^123^I‐labeled analogs and later with ^111^In‐ and ^99m^Tc‐labeled analogs, was commonly used for imaging of NENs, owing to their high affinity for SSTR2. However, due to some limitations (i.e., high physiological uptake such as in the liver, lack of detection of smaller lesions, low resolution of gammacameras) that may decrease the diagnostic efficacy, positron emission tomography (PET) with ^68^Ga‐1,4,7,10‐tetraazacyclododecane1,4,7,10‐tetraacetic acid (DOTA)‐conjugated peptides has largely replaced conventional scintigraphy, offering higher resolution, greater diagnostic accuracy, reduced radiation exposure, and improved patient compliance.^14^ The most commonly used ^68^Ga‐DOTA‐conjugated peptides are [^68^Ga‐DOTA^0^‐Tyr^3^] octreotide (^68^Ga‐DOTATOC), [^68^Ga‐DOTA^0^‐^1^NaI^3^]octreotide (^68^Ga‐DOTANOC) and [^68^Ga‐DOTA0‐Tyr^3^]octreotate (^68^Ga‐DOTATATE). These radiopharmaceuticals exhibit high affinity not only for SSTR2A, but also for SSTR5 (^68^Ga‐DOTATOC) and SSTR3/SSTR5 (^68^Ga‐DOTANOC).^15^, ^16^ In addition, ^64^Cu‐DOTATATE has also been used in NEN patients.^17^ Although these above‐mentioned radiopeptides function as receptor agonists for SSTR, some other radiopeptides with antagonist activity on SSTR have recently been proposed. In preliminary studies, these radiolabeled antagonists show a higher tumor uptake due to higher affinity binding to SSTR2 as compared to the agonists.^18^, ^19^

Along with SSTR, NENs can be imaged by targeting other molecular and metabolic pathways due to their NE functional features. In this scenario, catecholaminergic tracers (both gamma and beta emitters) such as ^123^I/^124^I‐metaiodobenzylguanidine (^123^I/^124^I‐mIBG), ^18^F‐3,4‐dihydroxyphenylalanine (^18^F‐DOPA), meta‐hydroxyephedrine (^11^C‐HED), and meta‐[^18^F]fluorobenzylguanidine ([^18^F]mFBG) can be used in tumors with low/variable SSTR expression and exploit their function in medullary thyroid cancer, midgut NEN, neuroblastoma (NB), or paraganglioma (PGL), and in patients with doubt for synchrone/metachrone metastatic malignancy (e.g., breast cancer).^16^

In addition, a number of other molecular imaging probes are in development, including radiolabeled glucagon‐like peptide 1 receptor ligands, which show particular promise in imaging insulinoma^20^; radiolabeled agonists interacting with the chemokine receptor, CXCR4, which is frequently overexpressed in high‐proliferating/advanced tumors, including small cell lung cancer (SCLC)^21^; ^68^Ga‐DOTA‐labeled cholecystokinin (CCK) 2 receptor ligands for imaging medullary thyroid cancer and SCLC^22^, ^23^; radiolabeled bombesin receptor (also known as gastrin‐releasing peptide receptor [GRPR ligands, agonists/antagonists]; BB1, BB2, and BB3 receptors) which can image a large range of tumors (prostate, colon, breast, central nervous system tumors, NETs)^24^; and radiolabeled ligands that interact with vasoactive intestinal peptide (VIP) and pituitary adenylate cyclase‐activating polypeptide (PACAP) receptors (VPAC1, VPAC2, PAC) and with the glucose‐dependent insulinotropic‐polypeptide receptor.^25^ In this scenario, fibroblast activation protein inhibitor used for PET imaging and targeting tumor microenvironment has also been investigated in NEN patients.

Finally, ^18^F‐2‐fluorodeoxyglucose (^18^F‐FDG) PET/CT plays a key role in the NENs scenario. In addition to its proven role as a prognostic marker, ^18^F‐FDG PET could provide important information on tumor heterogeneity, guide treatment decisions (i.e., combination/alternative treatment), and provide information on disease progression, thereby impacting patient management.

### 1.2 From imaging to treatment of NENs through different radioisotopes

PRRT with SSAs was first introduced into clinical practice in 1992 by the Rotterdam group, employing high activities of ^111^In‐pentetreotide.^26^ However, the therapeutic efficacy of this radiopharmaceutical was limited by the very low energy (<1 keV) released by the Auger‐emitter indium‐111 in a sphere of just a few cubic nanometers (with a range of about 80–200 nm). As a result, isotopes with higher energy and longer penetration range, such as Yttrium‐90, were introduced. The beta particles emitted by ^90^Y (maximum energy 2.27 MeV, maximum penetration range in soft tissue 11 mm, half‐life 64 h) enable the direct killing of SSTR‐positive cells while also producing a cross‐fire effect, targeting nearby receptor‐negative tumor cells. Additionally, since 2000, the chelate analog [DOTA0,Tyr3]‐octreotate (DOTA‐TATE), with sixfold‐ to ninefold higher affinity for SSTR2, was introduced. It can be labeled with the β‐γ emitter Lutetium‐177 (maximum energy β‐0.49 MeV, maximum penetration range β‐particle penetration depths in soft tissue 1.7 mm, half‐life 6.7 days), providing similar efficacy to ^90^Y‐labeled radiopharmaceuticals but with presumably lower toxicity, particularly to the kidneys.^1^ In recent years, the need to enhance PRRT outcomes has led to the development of targeted alpha therapy (TAT). These new radiopharmaceuticals emit high‐energy (5–9 MeV) but short‐range (40–100 μm) alpha particles, inducing cell death by double‐stranded DNA breaks, thereby minimizing systemic side effects. Various therapeutic radionuclides have been investigated, such as Bismuth‐213 (^213^Bi), Actinium‐225 (^225^Ac), Terbium‐149 (^149^Tb), and Lead‐212 (^212^Pb). This emerging therapeutic approach holds promise in overcoming the limitations of β‐emitting radiopharmaceuticals.^27^

## 2 SEARCH STRATEGY

A PubMed/MEDLINE and Google Scholar search of the published literature was performed using a combination of the search terms “peptide receptor radionuclide therapy,” “PRRT,” “neuroendocrine neoplasm,” “NEN,” “neuroendocrine tumor,” “^177^Lu‐DOTATATE,” “^90^Y‐DOTATOC,” “alpha emitters,” “pulmonary carcinoid,” “lung neuroendocrine,” “paraganglioma,” “pheochromocitoma,” “medullary thyroid cancer,” “meningioma,” “thyroid carcinoid,” “pulmonary neuroendocrine,” “medullary thyroid cancer,” “neuroblastoma,” “medulloblastoma,” “pituitary,” “Merkel cell carcinoma,” “SSTR‐targeted therapy,” until August 2024. Additional literature was retrieved from the reference lists of all identified articles. After screening titles and abstracts and reading full texts, only articles with more than 10 patients and with homogeneous treated cohort (per tumor type) were included. However, for tumors with less evidence available, smaller sample studies and case reports were also considered.

## 3 RESULTS

### 3.1 Lung NENs

Lung NENs are a heterogeneous group of pulmonary neoplasms showing NE morphology and immunophenotype. According to the 2021 World Health Organization (WHO) classification, lung NENs were divided into four entities: typical carcinoid (TC), atypical carcinoid (AC), small‐cell lung carcinoma (SCLC) and large‐cell neuroendocrine carcinoma (LCNEC).^36^ In addition, combined NE and non‐NE carcinomas also exist.^37^ Carcinoids correspond to well‐differentiated NENs and include low‐grade (i.e., TC) and intermediate‐grade tumors (i.e., AC), while NECs correspond to high‐grade carcinomas and include SCLC and LCNEC.^36^ Many efforts have been made to unify the lung NENs terminology with the WHO terminology for GEPNENs.^37^ Namely, despite the differences in the anatomical sites, these tumor entities belong to the same family and should theoretically follow the same management used for NENs.

It was reported that SSTR2A is the SSTR subtype most frequently expressed immunohistochemically in lung NENs (72%), followed by SSTR1 (63%), SSTR5 (40%), and SSTR3 (20%).^38^

The current guideline^39^ recommends cold SSAs as the first‐line treatment for lung carcinoids. Everolimus is advised as first‐line therapy for AC or as second‐line therapy for TC and progressive lung carcinoids following SSAs. In the RADIANT‐4 trial,^40^ 81% of patients receiving everolimus achieved disease stabilization according to RECIST 1.1, compared to 64% in the placebo group, with improved progression‐free survival (PFS) (11.0 months vs. 3.9 months). Alternative treatments include temozolomide ± capecitabine, IFN‐α, PRRT, and platinum‐based chemotherapy, though no clear consensus exists due to the lack of clinical trials.^39^

Lung NENs are often included in cohorts of patients treated with PRRT along with other GEPNENs,^41^ and only a few studies have focused exclusively on the role of PRRT in lung NENs (Table 1). In 2016, Mariniello et al. retrospectively studied one of the largest cohorts of 118 patients with unresectable/metastatic bronchopulmonary carcinoid who received ^177^Lu‐DOTATATE (*n* = 48), ^90^Y‐DOTATOC (*n* = 45), or a combination thereof (*n* = 21). Median progression‐free survival (mPFS) and median overall survival (mOS) were 28.0 and 58.8 months, respectively. Morphologic responses (partial responses + minor responses) were reached in 26.5% of patients and were associated with longer OS and PFS, with the combination of ^90^Y‐DOTATOC plus ^177^Lu‐DOTATATE protocol achieving the highest overall response rate (ORR, 38.1%).^28^ The study is, however, affected by a selection bias due to the inclusion of patients with different disease aggressiveness, along with the heterogeneity of the treatment scheme adopted. In addition, full biochemical data were not consistently available after PRRT, potentially missing toxicity assessment.^28^ In the same year, Ianniello and colleagues reported on 34 patients who received four/five cycles of ^177^Lu‐DOTATATE. The overall disease control rate (DCR) was 62%, the mPFS was 18.5 months (95% CI, 12.9–26.4 months) and the mOS was 48.6 months (95% CI, 26.4–68.9 months), resulting in a better mPFS (20.1 months vs. 15.7 months) and mOS (48.6 months vs. 37 months) in patients with TC compared to AC. No significant acute or delayed toxicities (CTCAE Grade 3 or 4) were reported.^29^ Conversely, in the study by Sabet et al. investigating 22 patients with metastatic, unresectable pulmonary NET who underwent PRRT with ^177^Lu‐DOTATATE (mean activity of 7.8 ± 0.68 GBq), relevant hematotoxicity (Grade 3) was observed in 3 patients (13.6%) at 3–10 weeks after at least one of the administrations, although no significant nephrotoxicity (≥ Grade 3) was observed during the follow‐up. Similar DCR (68.1%), mPFS (27 months, 95% CI, 9–45) and mOS (42 months, 95% CI, 25–59) were reported.^30^

**TABLE 1:** Characteristics of the included studies on lung neuroendocrine neoplasms.

Author, year (ref)	*n*	RF	Response (%)		Outcome (months)		Toxicity (G3/G4), *n*
			ORR	DCR	mPFS	mOS	
Mariniello et al., 2016^28^	118	^177^Lu‐DOTATATE ^90^Y‐DOTATOC	26.5	67.3	28.0	58.8	2 G3 anemia 3 G3 leucopenia 2 G3 thrombocytopenia
Ianniello et al., 2016^29^	34	^177^Lu‐DOTATATE	15	62	18.5	48.6	No
Sabet et al., 2017^30^	22	^177^Lu‐DOTATATE	27.3	68.2	27.0	42.0	3 G3 hematotoxicity (NS)
Mirvis et al., 2020^31^	25	^177^Lu‐DOTATATE ^90^Y‐DOTATOC	40	88	17.0	42.0	1 G3 thrombocytopenia 1 G3 pericarditis
Minutoli et al., 2021^32^	14	^177^Lu‐DOTATATE ^90^Y‐DOTATOC/TATE ^111^In‐Pentetreotide	21	71	—	—	No
Parghane et al., 2017^33^	22	^177^Lu‐DOTATATE	31 (RECIST 1.1) 37 (^18^F‐FDG + ^68^Ga‐DOTATOC)	68 (RECIST 1.1) 53 (^18^F‐FDG + ^68^Ga‐DOTATOC)	—	40.0	No hematotoxicity
Lim et al., 2020^34^	48	^177^Lu‐DOTATATE	33	83	—	49.0	2 G3‐5 hematotoxicity (NS) 1 G3 nausea 1 AML 1 acute kidney injury 1 superior vena cava obstruction
Zidan et al., 2022^35^	48	^177^Lu‐DOTATATE	20 (RECIST 1.1) 44 (^68^Ga‐DOTATOC)	88 (RECIST 1.1) 88 (^68^Ga‐DOTATOC)	23.0	59.0	1 G3 leucopenia 6 G3 lymphopenia

Abbreviations: AML, acute myeloid leukemia; DCR, disease control rate; mOS, median overall survival; mPFS, median progression‐free survival; NS, not specified; ORR, overall response rate.

Regarding the clinical response profile, single‐center^31^, ^32^, ^33^ or multicenter^34^ studies reported significant improvement of symptoms in more than half of treated patients. Specifically, in the Australian multicenter study, the authors showed a significant benefit when considering both patients with secretory symptoms, such as flushing and diarrhea, and those with non‐secretory symptoms, including pain and dyspnea.^33^ Similarly, Parghane et al. retrospectively analyzed 22 patients with symptomatic disease prior to PRRT. They reported symptomatic response in up to 79% of patients. In addition, a biochemical response was also demonstrated in 53% of cases.^33^ However, symptom evaluation was based on retrospective review of medical records, and quality of life (QOL) questionnaires were not used, limiting the generalizability of results.

Response assessment in NEN is currently controversial due to the lack of standardized methods for assessing response to PRRT,^42^ leading to significant differences in the criteria used between studies. Several authors observed a discrepancy between morphological (i.e., computed tomography [CT]) and metabolic (i.e., ^68^Ga‐DOTATOC and/or ^18^F‐FDG PET/CT) imaging when assessing response in the same group of patients.^33^, ^35^

The reliability of the conclusions is limited by the small sample size and the retrospective design of most of the included studies. Moreover, the variability in disease aggressiveness, differences in treatment approaches (^90^Y‐ and/or ^177^Lu‐based PRRT), and previous and/or simultaneous treatments varied greatly with regard to type, modality, and timing across study cohorts. In addition, comparisons within studies should be approached with caution, as treatment protocols, dosages, and fractionation strategies were largely empirical and varied not only between studies but also within each patient cohort.

### 3.2 Metastatic PGL

PGL belongs to non‐epithelial NEN characterized by a strong genetic predisposition, involving mutations in more than 20 different genes, either germline or sporadic.^54^, ^55^ Among these, germline mutations in the succinate dehydrogenase enzyme complex (SDHx) genes are among the most common causes of PGL, occurring in up to 25% of cases.^56^, ^57^ In 2022, the WHO classification designated pheochromocytoma (PCC) as a subtype of intra‐adrenal PGL, originating from chromaffin cells in the adrenal medulla, accounting for 80%–85% of neural crest‐derived tumors. The remaining 15%–20% includes sympathetic abdominal PGL, sympathetic head and neck PGL, and parasympathetic PGL.^58^

The therapeutic strategy for metastatic PGL is primarily aimed at controlling excessive catecholamine secretion and tumor burden, as there are no curative treatment options. Therapeutic alternatives include watch‐and‐wait (including alpha‐blockers to manage hypertension), locoregional therapies, radionuclide treatment, systemic chemotherapy, and molecular targeted therapies.^59^ Historically, the treatment of metastatic PGL with radionuclide therapy has primarily involved the ^123^I‐MIBG/^131^I‐MIBG thera(g)nostic pair, with clinical trials reporting objective responses in 23% of cases according to the RECIST.^60^, ^61^

**FIGURE 1:**
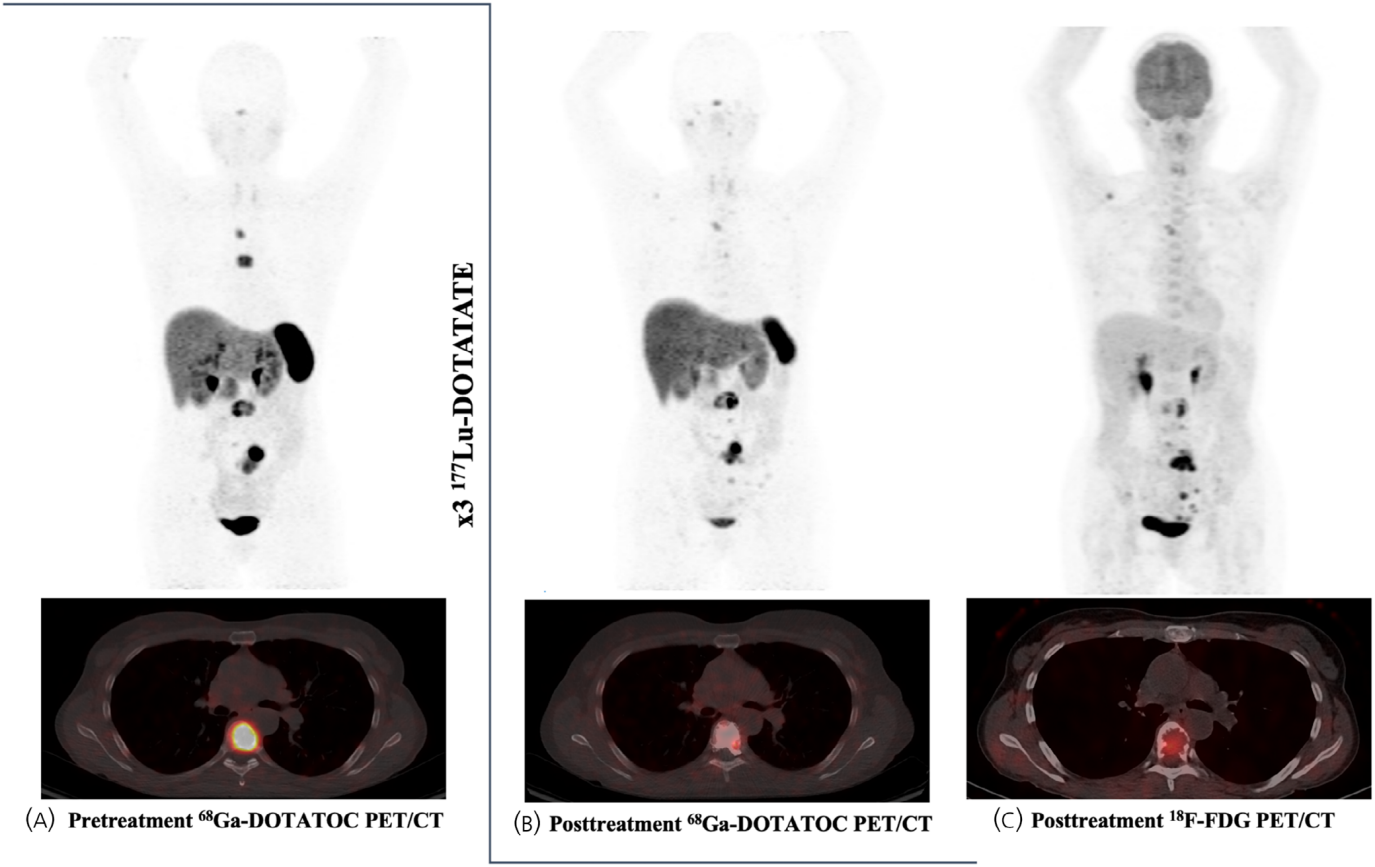
A 52‐year‐old female patient with metastatic pheochromocytoma treated with ^177^Lu‐DOTATATE. ^68^Ga‐DOTATOC PET/CT study prior to (A) and after (B) three cycles of ^177^Lu‐DOTATATE treatment (cumulated activity 21.30 GBq). The post‐treatment ^18^F‐FDG PET/CT (C) confirmed the sites of the disease and showed an overall partial response to treatment. The patient progressed 14 months after the end of PRRT and died 20 months after the end of treatment. PET, positron emission tomography; PRRT, peptide receptor radionuclide therapy.

However, it has also been demonstrated that PGLs commonly overexpress SSTRs, predominantly SSTR2,^4^, ^55^ particularly those pseudohypoxic subtypes related to tricarboxylic acid cycle mutations,^62^ supporting the thera(g)nostic use of SSA (Table 2 and Figure 1). Comparisons between different radionuclide therapies, including ^90^Y‐DOTATATE, ^177^Lu‐DOTATATE, and ^131^I‐MIBG, did not show a clear advantage of one radiopharmaceutical over the others.^44^, ^52^ However, receptor expression varies between patients, and up to 50% of patients have been shown to be ineligible for both therapies.^52^ Thus, the treatment strategy in PGL (as well as for other NEN tumors) should always be guided by the extent and intensity of disease defined through pre‐therapeutic ^123^I‐MIBG and SSTR imaging.

**TABLE 2:** PGL and PRRT (^177^Lu‐/^90^Y‐DOTA‐TATE/TOC).

Author, year (ref)	Patients	Disease control	Treatment cycles	Median PFS (months)
Forrer et al., 2008^43^	28	20/28 (71%)	1–4	‐
Nastos et al., 2017^44^	13	13/13 (100%)	1–4	38.5
Kong et al., 2017^45^	20	15/17 (88%)	1–4	39.0
Zandee et al., 2019^46^	30	27/30 (90%)	4	30.0
Vyakaranam et al., 2019^47^	22	2/22 (100%)	3–11	21.6
Kolasinska‐Cwikla et al., 2019^48^	13	10/12 (83%)	2–5	35.0
Jaiswal et al., 2020^49^	15	12/15 (80%)	1–6	Not reached
Severi et al., 2021^50^	47	37/46 (80%)	5	Not reached
Nilica et al., 2021^51^	19	8/16 (50%)	2–5	96.0
Prado‐Wohlwend et al., 2022^52^	17	9/10 (88%)	1–4	29.0
Rubino et al., 2024^53^	30	26/30 (87%)	2–8	5‐y PFS: 68% (95% CI, 48–82)

Abbreviations: PFS, progression‐free survival; PGL, paraganglioma; PRRT, peptide receptor radionuclide therapy.

As regards PRRT alone, in 2008 Forrer et al. reported on 28 patients with inoperable PGL, including 9 with PCC and 19 with non‐surgically curable PGL, treated with up to 4 cycles of ^90^Y/^177^Lu‐DOTATOC. Among the 26 patients who completed treatment, 2 showed PR, 5 had minor response (MR), and 13 achieved SD, resulting in a DCR of 77%. The treatment was well tolerated, with no serious adverse events reported.^43^ Concerning symptomatic and biochemical responses to treatment, a bicentric study from Australia reviewed 20 patients with unresectable PGL treated with ^177^Lu‐DOTATATE, receiving a median cumulative activity of 22 GBq (1–4 cycles). Nine patients also received radiosensitizing chemotherapy. After treatment, 62% of patients could de‐escalate medications for symptom management, and 86% showed a reduction in CgA levels, with 58% experiencing a reduction of more than 50%. A decrease in plasma metadrenaline and normetadrenaline levels occurred in most patients with secondary hypertension. Although the biochemical marker CgA was assessed in most patients, serum or urine catecholamine levels were not routinely measured in one of the two centers involved, so these results should be taken with caution. In the same study, the authors reported Grade 2 lymphopenia as the most common toxicity, while four patients developed Grade 3 lymphopenia and two had Grade 3 thrombocytopenia. One patient with pre‐existing renal impairment experienced further deterioration, though no dialysis was required, and another patient with chronic renal failure had a decline in renal function due to systemic amyloidosis rather than PRRT. No additional toxicities were seen in patients who received radiosensitizing chemotherapy; however, any additional effectiveness of the combined treatment was difficult to prove due to the limited number of patients.^45^ In a separate 2019 study by the Rotterdam group involving 30 patients (17 parasympathetic PGL, 10 sympathetic PGL, 3 metastatic PCC), Grades 3–4 subacute hematotoxicity occurred in 20% of patients, and 1 developed myelodysplastic syndrome after 6 cycles of ^177^Lu‐DOTATATE.^46^ Conversely, Vyakaranam et al. in 22 patients (13 PCCs, 9 PGLs) treated with ^177^Lu‐DOTATATE (median activity 29.6 GBq) showed that, though common (73%), hematological side effects were generally mild (Grades 1–2); however, with the limitation of a short follow‐up (up to 3 months after the last treatment). The authors also reported a mOS of 49.6 months, and mPFS of 21.6 months, with 2 patients achieving PR, while 20 achieved SD. Biochemical responses were >50% catecholamine reduction in 25% and >50% CgA reduction in 40% of patients, respectively. Longer OS and PFS were observed in patients with Ki‐67 <15% and in those receiving PRRT as first‐line therapy.^47^ Among other predictive factors of PRRT response, germline mutations in SDHD or SDHB genes were also studied.^48^ To note, there are five PGL–PCC syndromes associated with heterozygous germline mutations in genes encoding the subunits of the succinate dehydrogenase enzyme complex.^63^ In a prospective, single‐institution open‐label, Phase II study on 13 PGL patients treated with ^90^Y‐DOTATATE, the authors reported significant differences in terms of mOS and mPFS between patients with PGL1–*SDHD* gene mutation compared to subjects with PGL4–*SDHB* gene mutation (*p* = 0.05 and *p* = 0.014, respectively).^48^ In addition, the baseline ^68^Ga‐DOTATOC SUV_max_ also showed to be significantly associated with response with the area under the curve of 0.939 (*p* = 0.024). Namely, a SUV_max_ of >21 had a sensitivity of 0.91 (95% CI, 0.80–1.00) and specificity of 1.0 (95% CI, 0.29–1.00) to predict response to PRRT.^49^

In 2021, Severi et al. prospectively enrolled 47 patients with metastatic PGLs, treated with ^90^Y‐DOTATOC (*n* = 12, median cumulative activity 9.2 GBq) or with ^177^Lu‐DOTATATE (*n* = 34, median cumulative activity 24.42 GBq). The authors demonstrated a better mOS with ^177^Lu‐DOTATATE compared to ^90^Y‐DOTATOC (143 months vs. 92 months), probably due to the higher affinity of tyr3‐octreotate for SSTR2 receptors, with a consequent longer tumor residence time of ^177^Lu‐DOTATATE. The DCR was higher in patients without risk factors (overall DCR ~80%) compared to those treated with reduced cumulative activity who presented risk factors for bone marrow and/or renal toxicity (i.e., DCR 33.3% and 55% for patients treated with ^90^Y‐DOTATOC and ^177^Lu‐DOTATATE, respectively).^50^ In a dosimetry‐based study, Nilica et al. showed that from 19 patients with PGL, 13 patients received ^90^Y‐DOTATOC PRRT in 1–4 cycles. The estimated tumor dose ranged between 4.6 and 152.8 Gy (mean 51.1 Gy, median 38.3 Gy) resulting in a stabilization of the disease in most patients after three therapy cycles with approximately 3.7 GBq ^90^Y‐DOTATOC each.^51^ Recently, Rubino et al. published retrospective data on 22 PGL and 8 PCC patients followed up for 8.9 years. The 5‐year and 10‐year PFS was 68% (95% CI, 48–82) and 53% (95% CI, 33–69), respectively. The 5‐year and 10‐year OS was 75% (95% CI, 54–87) and 59% (95% CI, 38–75), respectively. Persistent late toxicity occurred in one patient (G3 anemia; G2 leucopenia). Only 3 patients developed persistent mild chronic kidney disease after treatment.^53^ At the time of writing, the National Cancer Institute is sponsoring an open‐label, single‐arm, multicenter Phase II study evaluating the efficacy and safety of ^177^Lu‐DOTATATE in SSTR‐positive PGL/PCC (NCT03206060). Results of the study are expected in 2027.

### 3.3 Medullary thyroid carcinoma

Medullary thyroid carcinoma (MTC) is a NE/endocrine tumor originating from the neural crest and arising from the parafollicular C‐cells. About 80% of cases are sporadic, while the rest are divided into three familial forms: multiple endocrine neoplasia (MEN) Type 2A, MEN Type 2B, and familial MTC not associated with MEN.^69^

Currently, the treatment of advanced or metastatic MTC primarily consists of cabozantinib and vandetanib, two antiangiogenic multikinase inhibitors (MKIs).^70^ These therapies offer an objective response rate of up to 45% and have been demonstrated to improve PFS and OS compared to placebo.^71^ There is limited evidence supporting the use of chemotherapy or radionuclide therapy in MTC patients; however, these options may be considered when MKIs are contraindicated.^70^

In vitro data demonstrated SSTR expression by MTC cells in more than 75% of cases (SSTR2 91.6%, SSTR5 75%, SSTR3 41.6%, and SSTR1 33.3%),^72^ later confirmed by in vivo analysis using SSTR‐based imaging.^73^ Since the early 2000s, some clinical experience with Auger emitters has been reported, though it remains limited to case studies.^74^, ^75^ Most available evidence is based on beta emitters, but studies involving more than 10 patients are still scarce (Table 3). A meta‐analysis published in 2020 evaluating a total of 98 patients with MTC treated with various PRRT schemes showed an ORR of 8.5% (95% CI, 1.9%–19.2%), while a DCR of 60% (95% CI, 49.6%–69.8%), with a 2.8% rate of serious adverse events.^76^ The therapeutic efficacy of ^90^Y‐DOTATOC was retrospectively evaluated by Bodei et al. in 21 metastatic MTC treated with a median cumulative activity of 10.4 GBq (range, 7.5–19.2) given in 4 cycles (range, 2–8). Most of these patients had previously been treated only with cold SSAs; however, the cohort also included patients treated with multiple lines of therapy prior to ^90^Y‐DOTATOC. The treatment was well tolerated, with only one case of Grade 3 hematological toxicity. Morphological CR was documented only in two patients, and no PR was observed. Biochemical evaluation (i.e., calcitonin and CEA) confirmed progression in 57% of patients.^64^ The Basel group in a Phase II, single‐center, open‐label trial investigated the response, survival, and safety profile of ^90^Y‐DOTATOC in 31 progressive metastatic MTC. A post‐therapeutic prolongation of calcitonin doubling time of at least 100% was found in 18 of 31 participants (58.1%). Among these, decreasing calcitonin levels were found in nine patients (29.0%) following treatment. In responders, the median reduction of serum calcitonin was 45.2% (range, 0.4%–96.3%). The mOS was 91 months (range, 2.2–373.1 months) from the time of diagnosis and 15.7 months (range, 1.4–107.0 months) from the time of the first ^90^Y‐DOTATOC treatment, with responders showing a significantly longer mOS as compared with nonresponders. In addition, higher cumulative activities of ^90^Y‐DOTATOC were associated with a nonsignificant trend toward longer survival outcomes. Only one case (3.2%) of Grade 3 acute, transient thrombocytopenia was observed. However, six patients (19.4%) experienced renal toxicity of any grade, with one case of Grade 4 renal toxicity occurring 25.8 months after therapy. These results, though promising, were derived from a single study cohort. In addition, the authors were not able to define pretherapeutic markers of post‐therapeutic response.^65^ As regards prognostication of response to therapy, another study found high uptake on the ^111^In‐DTPA‐octreotide scans (uptake grade ≥3) and moderate‐to‐positive SSTR2A receptor expression on histological examination to be associated with DCR after treatment.^66^

**TABLE 3:** ^177^Lu‐/^90^Y‐DOTA‐TATE/TOC in medullary thyroid cancer.

Author, year (ref)	*n*	RF	Response (%)		Outcome (months)	
			ORR	DCR	mPFS	mOS
Bodei et al., 2004^64^	21	^90^Y‐DOTATOC	10	67	n.a.	n.a.
Iten et al., 2007^65^	31	^90^Y‐DOTATOC	‐	n.a.	n.a.	91.0
Beukhof et al., 2019^66^	10	^177^Lu‐DOTATATE	/	40	8.4	26.0
Parghane et al., 2020^67^	43	^177^Lu‐DOTATATE	4 (RECIST 1.1) 10 (^18^F‐FDG + ^68^Ga‐DOTATOC)	58 (RECIST 1.1) 61 (^18^F‐FDG + ^68^Ga‐DOTATOC)	24.0	26.0
Liu et al., 2023^68^	28	^177^Lu‐DOTATATE ^90^Y‐DOTATOC	12	56	10.1	63.7

Abbreviations: DCR:disease control rate; mOS, median overall survival; mPFS, median progression‐free survival; n.a., not assessed; ORR, overall response rate.

Most recently, Parghane et al. investigated 43 patients with SSTR‐positive metastatic MTC, who received PRRT with ^177^Lu‐DOTATATE. Out of 43 patients, two had a positive family history of MTC and were diagnosed with MEN2A. Of those patients with MEN2A, one had undergone surgical excision of PCC and parathyroid glands, and 12/43 had received EBRT before PRRT. All patients were symptomatic before the start of PRRT. The mPFS was 24 months (95% CI, 15.1–32.9 months) from the time of the first ^177^Lu‐DOTATATE treatment. A significantly longer PFS from the time of the first ^177^Lu‐DOTATATE treatment was found in patients with a calcitonin doubling time (CtnDT) of more than 24 months as compared to patients with less than 24 months CtnDT (mPFS not reached vs. 10 months, *p* < 0.001). The mOS was 26 months (95% CI, 16.6–35.3 months) from the time of the first ^177^Lu‐DOTATATE treatment. Similarly, patients having more than 24 months CtnDT had a significantly longer OS as compared to patients with less than 24 months CtnDT (mOS 60 months vs. 20 months, *p* < 0.001). Based on symptomatic and biochemical response evaluation criteria, 22/43 patients were responders (51%) and 21/43 were nonresponders (49%) to PRRT. Based on RECIST 1.1 criteria, 2/43 (4%) patients had PR, 25/43 (58%) patients had SD, and 16/43 (38%) patients had PD. However, differences in tumor burden prior to PRRT and inter‐ and intra‐patient variation in FDG uptake may reflect differences in tumor aggressiveness among the patients included in the study.^67^ In 2023, Liu et al. reported on 28 patients with progressive, SSTR‐positive advanced MTC who received PRRT with ^177^Lu‐ or ^90^Y‐labeled SSAs. The authors also identified the presence of bone metastases as a significant prognostic factor associated with poor OS and PFS.^68^

### 3.4 Progressive/metastatic meningioma

Meningiomas originate from the arachnoid cells located on the inner surface of the dura, with the latter originating from meningeal precursor cells derived from mesoderm and neural crest.^83^, ^84^ The WHO classification distinguishes meningiomas into three grades, according to different histological patterns: low‐grade meningioma (WHO‐I) and high‐grade meningioma (WHO‐II and WHO‐III).^85^

Inoperable or recurrent tumors can be treated with radiosurgery or with fractionated radiotherapy.^86^ Because of their limited efficacy, systemic therapies are chosen on an individual basis once surgical and radiation possibilities have been exhausted.^86^, ^87^

Based on the high density of SSTR (especially Type 2) in more than ~90% of meningiomas, diagnostics and therapy (thera(g)nostics) of this tumor entity can be obtained with radiolabeled SSAs.^88^, ^89^, ^90^ Also in this setting, most of the available literature is based on beta emitters (Table 4)^77^, ^78^, ^79^, ^80^, ^81^, ^82^ although some groups have also used the auger emitter In‐111.^91^ In a meta‐analysis of 111 patients, DCR was achieved in 63% of cases. The 6‐month PFS rates were 94%, 48%, and 0% for Grades I, II, and III, respectively. The 1‐year OS rates were 88%, 71%, and 52% for Grades I, II, and III, respectively.^92^ In 2009, Bartolomei et al. evaluated 29 meningioma patients who underwent ^90^Y‐DOTATOC for 2–6 cycles for a cumulative activity of 5–15 GBq. Post‐treatment MRI performed 3 months after completion of treatment showed disease stabilization in 19 of 29 patients (66%) and progressive disease in the remaining 10 (34%). Better results were obtained in patients with Grade I meningiomas than in those with Grades II–III, with a median time to progression of 61 months in the low‐grade group and 13 months in the high‐grade group.^77^ Similarly, Marincek et al. reported on 34 patients treated with ^90^Y‐ and/or ^177^Lu‐DOTATOC. A total of 74 treatment cycles (1–4 cycles per patient) were performed, including 66 cycles of ^90^Y‐DOTATOC (range, 1.5–18.3 GBq) and 8 cycles of ^177^Lu‐DOTATOC (range, 7.4–22.2 GBq). Disease stabilization was achieved in 23 treated patients (66%), who also experienced prolonged survival compared to PD patients. However, severe hematotoxicity (three patients) and severe renal toxicity (one patient) were also reported.^79^ A higher rate of disease control (86.7%) was shown by Gerster‐Gilliéron et al. in 15 patients with recurrent or progressive meningiomas treated with systemic ^90^Y‐DOTATOC (7.4 MBq/m^2^ in 2 fractions) with no Grade 4 toxicities reported. However, the study included 60% of patients with low‐grade meningiomas.^80^ The main side effects reported by other authors are related to transient hematotoxicity, such as anemia (22%), leukopenia (13%), lymphocytopenia (24%), and thrombocytopenia (17%).^92^ In this regard, Minczeles et al. in 15 meningioma patients treated with ^177^Lu‐DOTATATE (administered activity of 7.4 GBq/cycle up to 4 cycles) reported at least one type of subacute hepatic or hematologic toxicity in 13/15 (87%) patients: Grade 1 in 10 (67%) patients, Grade 2 in 4 (27%) patients, Grade 3 in 8 (53%) patients, and Grade 4 in 1 (7%) patient. Severe Grade 3 or 4 toxicity consisted only of lymphocytopenia.^81^ Lymphocytopenia was also confirmed by other authors as the most common adverse event.^82^ Considering that absorbed dose estimations at the organs at risk were largely within safety limits,^93^, ^94^, ^95^ it is expected that treatment schedules could be significantly optimized based on dosimetry.^96^

**TABLE 4:** Meningioma (^177^Lu‐/^90^Y‐DOTATATE/TOC).

Author, year (ref)	Patients	Disease control	Treatment cycles	Median PFS (months)
Bartolomei et al., 2009^77^	29	19/29 (66%)	2–6	6.0
Gerster‐Gilliéron et al., 2015^78^	15	13/15 (86.7%)	2	24.0
Marincek et al., 2015^79^	34	23/34 (65.6%)	1–4	‐
Seystahl et al., 2016^80^	20	10/20 (50%)	3	5.4
Minczeles et al., 2023^81^	15	6/15 (40%)	1–4	7.8
Kurz et al., 2024^82^	14	9/14 (64%)	1–4	8.2

In the current evolving scenario, it is important to interpret existing data cautiously, as significant changes in tumor grading and classification over recent decades limit the applicability of previous findings to the current molecular diagnostic framework.^97^ Moreover, response assessment has been highly heterogeneous across published studies. The typically slow growth rate of meningiomas further complicates response evaluation, especially considering the short follow‐up periods in many studies.^97^ Recently, the RANO working group proposed standardized MRI‐based response criteria.^98^ In addition, although a framework for PET‐based response assessment is not yet available, the joint EANM/EANO/RANO/SNMMI practice guideline recommends using SSTR‐based imaging alongside MRI.^88^

**FIGURE 2:**
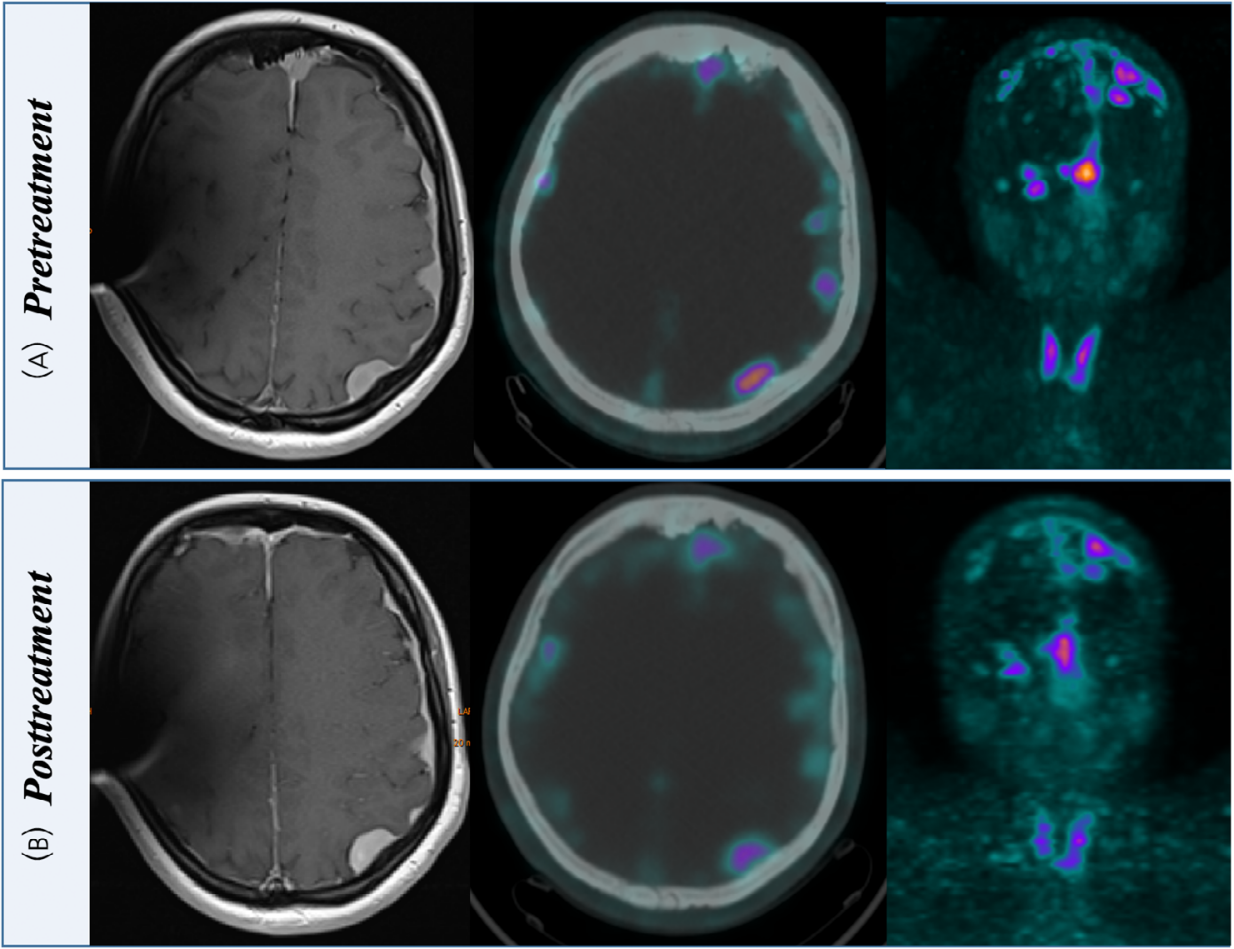
A 29‐year‐old patient suffering from neurofibromatosis Type II with multiple meningiomas and vestibular schwannoma. The patient underwent four cycles of ^177^Lu‐DOTATATE with a total administered activity of 29.97 GBq until April 2023. Pre‐treatment MRI and ^68^Ga‐DOTATOC PET/CT (A) showed multiple meningiomas, mostly supratentorial. Post‐treatment images (B) showed a minor response to PRRT. Fourteen months after the last course of therapy, the patient is still stable on imaging evaluation. PET, positron emission tomography; PRRT, peptide receptor radionuclide therapy.

The first randomized clinical trial comparing ^177^Lu‐DOTATATE with standard of care (LUMEN, NCT06326190) has recently begun recruitment, and results are eagerly awaited. Figure 2 shows a case example of a patient with multiple meningiomas treated with PRRT.

### 3.5 Radioiodine refractory differentiated thyroid carcinoma

Radioiodine therapy is one of the cornerstones in the management of differentiated thyroid carcinoma (DTC) following surgery; however, 5%–15% of patients become refractory to RAI, with a poor prognosis.^70^ Based on SELECT^99^ and DECISION^100^ trials, lenvatinib and sorafenib (i.e., MKIs drugs) are considered the standard first‐line systemic therapy for radioiodine refractory DTC (RAIR‐DTC).^99^

From the early 1990s, SSTR2 and SSTR5 expression was demonstrated in DTC, as well as in normal thyroid tissue.^101^, ^102^ SSTR2 is the predominant subtype in thyroid epithelial tumors with a high expression pattern, in particular, in papillary thyroid carcinoma (PTC).^103^ Compared to MTC and other neural crest‐derived carcinomas, limited data exist on the use of PRRT in RAIR‐DTC. In 2005, Gabriel et al. reported on five patients with recurrent/persistent DTC who received three/four cycles (1850 MBq/cycle) of ^90^Y‐DOTATOC. All patients presented with metastatic disease with more than one site affected, and stabilization of the disease was achieved for at least 5 months in all patients.^104^ Furthermore, in 2018, Roll et al. evaluated five patients with RAIR‐DTC who underwent ^177^Lu‐DOTATATE therapy (mean injected activity: 7.0 ± 0.7 GBq). After 2–4 therapy cycles, only one patient showed PR, whereas the remaining four progressed.^105^ A previous study by Czepczyński et al. reported on ^90^Y‐DOTATOC (total activity of 14.8 GBq in 4 therapy cycles) administered to six patients with RAIR‐DTC. Morphological response evaluated 3 months after the last treatment using RECIST criteria showed PR in one patient, SD in two patients, and PD in three patients. Biochemical response based on Tg measurements showed PR in one patient in agreement with imaging results, SD in four patients, and PD in one patient. However, most patients progressed within 1 year following PRRT. The mOS was 21 months from the first course of PRRT. No high‐grade hematological or renal toxicities were reported.^106^ Versari et al. prospectively enrolled 41 patients with progressive RAIR‐DTC, 11 of whom were also treated with PRRT receiving a fractionated injection of 1.5–3.7 GBq ^90^Y‐DOTATOC/administration. The response assessed by ^68^Ga‐DOTATOC PET/CT performed 3 months after the end of treatment was PR in 2/11 patients, SD in 5/11, while PD was observed in 4 cases.^107^ In a clinical Phase II, single‐center, open‐label trial, Iten et al. investigated response, survival, and the safety profile of ^90^Y‐DOTATOC treatment in 24 progressive RAIR‐DTC patients. A total of 58 cycles of ^90^Y‐DOTATOC (1–4 cycles per patient) were performed with a median cumulative activity of 13.0 GBq (range, 5.6–30.3 GBq). Decreasing Tg levels upon treatment with ^90^Y‐DOTATOC were found in seven (29.2%) patients. In these responders, the median reduction of serum Tg was 48.9% (range, 0.3%–54.5%), whereas in the nonresponders, the median increase of serum thyroglobulin was 36.8% (range, 2.6%–4912.8%). The mOS was 33.4 months (range, 3.6–126.8 months) from the time of diagnosis and 16.8 months (range, 1.8–99.1 months) from the time of the first ^90^Y‐DOTATOC treatment. Regarding the safety profile, eight (33.3%) patients developed hematologic toxicity: two (8.3%) patients developed acute transient leucopenia (one Grade 1 and one Grade 2), three (12.5%) patients developed acute transient thrombocytopenia (one Grade 1 and two Grade 3) and three (12.5%) patients developed anemia Grade 1. Four (16.7%) patients experienced permanent renal toxicity (two Grade 1, one Grade 2, and one Grade 4) at 3, 6, 7, and 11 months after therapy.^108^ To the best of our knowledge, neither the subsequent Phase III study has been published nor are there any clinical trials actively recruiting patients with RAIR‐DTC to undergo PRRT. Overall, in the systematic review by Lee et al., the authors reported a pooled proportion of patients with ORR of 15.61% (95% CI, 7.80%–26.74%) and DCR of 53.95% (95% CI, 41.13%–66.39%), with a pooled proportion of serious adverse events of 2.82% (95% CI, 0.03%–17.61%).^76^

In summary, the published studies are insufficient to establish the efficacy of PRRT in patients with RAIR‐DTC due to the small and heterogeneous patient cohorts. Based on available evidence, the efficacy of PRRT in these patients is limited and referral of patients to MKIs drugs has been favored. It should also be noted that most patients did not complete all treatment cycles, so it is possible that the dose to the tumor was not sufficient to ensure response.

### 3.6 Pediatric tumors: NB/medulloblastoma

**FIGURE 3:**
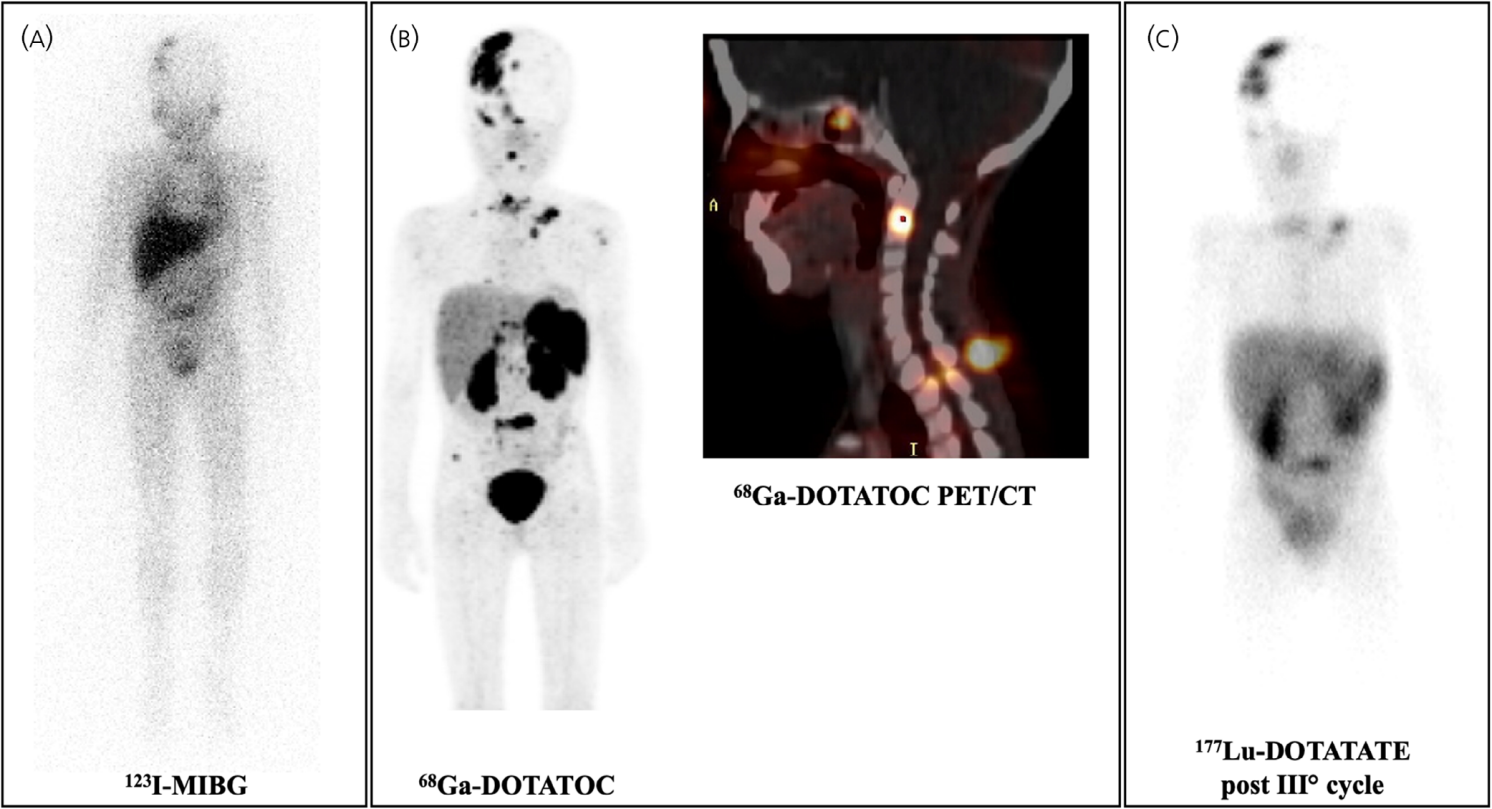
Pretreatment ^123^I‐MIBG (A) and ^68^Ga‐DOTATOC (B) before treatment with ^177^Lu‐DOTATATE (C) in a patient with neuroblastoma. A 9‐year‐old female patient with left paravertebral neuroblastoma was diagnosed in 2004. The patient underwent multiple lines of chemotherapy, tumor resection, antibody, angiogenesis inhibitor therapy, and external beam radiotherapy. Following tumor recurrence, she received an HLA bone marrow transplant and a total of 4 cycles of ^177^Lu‐DOTA‐TATE therapy with a total administered activity of 6.96 GBq. However, the patient progressed and died 6 months after the end of treatment.

NB is a neural crest‐derived malignancy of the peripheral nervous system and the third most common childhood tumor after leukemia and brain tumors.^109^, ^110^ The use of PRRT has been investigated in the treatment of NB, which often expresses SSTR.^111^, ^112^ Namely, various studies based on immunohistochemistry demonstrated the expression of all SSTR1 to SSTR5 on primary NB and, specifically, the overexpression of SSTR2 in the majority of NBs, even in recurrent/refractory tumor disease.^113^, ^114^ In 2011, Grains et al. first published results on six children with advanced (Stages 3–4) NB treated with two to three cycles of ^177^Lu‐DOTATATE therapy (4.04–7.5 GBq/kg, 8–10 weeks apart). According to RECIST, five patients obtained SD and one PD. Thrombocytopenia was the most frequent adverse event (*n* = 3 Grade 3, *n* = 1 Grade 4).^115^ Kong and colleagues in 2015 investigated four patients (3–9 years old) who received PRRT (^111^In‐DOTATATE, ^177^Lu‐DOTATATE, ^90^Y‐DOTATATE or combined). Toxicities were mainly hematological (i.e., thrombocytopenia, *n* = 1 Grade 4; anemia, *n* = 1 Grade 4 and *n* = 1 pancytopenia) occurring in patients heavily pretreated. One patient experienced an early clinical response to ^111^In‐DOTATATE treatment at the 3‐month assessment, followed by bone progression. In the absence of other therapeutic options, further PRRT was given (one cycle of combined ^111^In/^177^Lu‐DOTATATE, plus one cycle of ^177^Lu‐DOTATATE with oral temozolomide). The other patient had a favorable PR 3 months after the induction of ^111^In‐DOTATATE therapy, with sustained stability on ^68^Ga‐DOTATATE PET/CT, and remained clinically stable for 14 months after the first treatment. At progression, further PRRT was administered, including two cycles of ^177^Lu‐DOTATATE (second cycle with oral etoposide) with almost complete normalization of PET/CT at 3 months. This patient again had imaging relapse and received further ^177^Lu‐DOTATATE with temozolomide, resulting in a favorable partial response. However, there was evidence of bone marrow progression 2 months later with right thigh pain, and the augmentation of PRRT with further cycles of ^177^Lu‐DOTATATE (4 GBq) with ^90^Y‐DOTATATE (1 GBq) was selected rather than combining with chemotherapy. Two patients died due to progressive disease. The authors estimated a median best PFS for PRRT of approximately 10.5 months (2–20 months).^116^ Ten years after the study by Grains et al., the results of the LuDo phase IIa trial were then published by the same group.^117^ Twenty‐one patients with histologically confirmed relapsed or refractory metastatic high‐risk NB were included. Of the 21 registered, 20 received at least one course of ^177^Lu‐DOTATATE treatment, with only 8 patients undergoing the full 4 courses of ^177^Lu‐DOTATATE. There was no treatment‐related mortality; however, 10 serious adverse events were reported in 6 patients, and 1 case of dose‐limiting hematological toxicity was recorded. The median whole‐body radiation absorbed dose was 0.24 Gy (range, 0.14–0.42 Gy) following the first course when the administered activity was 75 MBq/kg, and 0.33 Gy (range, 0.16–0.61 Gy) following subsequent courses when the administered activity was 100 MBq/kg. The median cumulative whole‐body absorbed dose in those patients who received all four courses for whom complete data were available was 1.26 Gy (range, 0.97–1.48 Gy). The median cumulative mean renal radiation dose in the six patients who received four courses of treatment and for whom full dosimetric data were recorded was 16.5 Gy (range, 9.5–21.5 Gy). In 24 measurable lesions, the median tumor dose per course was 2.43 Gy (range, 0.04–13.50 Gy). Of the 20 treated patients, 6 died before response assessment, and none of the remaining 14 evaluable patients showed a response to treatment either by the original or by the revised International Neuroblastoma Response Criteria at 1 month after completion of treatment. In view of this, the trial was closed prematurely. The PFS at 6 months was 38% (95% CI, 18%–58%) and PFS at 12 months was 5% (95% CI, 0%–20%). The OS at 6 months was 62% (95% CI, 38%–79%) and OS at 12 months was 52% (95% CI, 30%–71%). Figure 3 shows a case example of a NB patient treated with PRRT.

Despite the low efficacy provided by the LuDo trial, three new trials are still recruiting. The LuDO‐N trial (NCT04903899, https://clinicaltrials.gov/ct2/show/NCT04903899) is planned to establish whether ^177^Lu‐DOTATATE can be effective as a single agent in the treatment of relapsed or primary refractory high‐risk NB when the administration schedule is intensified to two infusions delivered 2 weeks apart and the administered activity is personalized by dosimetry. The NEUROBLU 02 (NCT03966651, https://clinicaltrials.gov/ct2/show/NCT03966651) trial uses a dose escalation design to assess the effective dose and the highest dose of ^177^Lu‐DOTATATE that can be given safely without the need for stem cell re‐infusion. In order to make results from the NEUROBLU 2 and LuDO‐N trials comparable, the dosimetry and response evaluation protocols of these two trials have been harmonized. Another similar multi‐center trial in the United States is already recruiting (NCT04023331). Differently from the previous one, the latter will use the SARTATE, which binds to the SSTR‐2 labeled with ^67^Cu (β‐emitters).

In addition to NB, the expression of SSTR was also documented in medulloblastoma, the most common malignant intracranial tumor in children.^5^ However, clinical experience on the use of PRRT is only anecdotal.^118^

### 3.7 Pituitary neuroendocrine tumors

First identified as pituitary adenoma, the 2021 WHO classifications categorized this entity as a NET and proposed the name to be revised to pituitary NET (PitNET).^119^ Pituitary NET originates from the adenohypophysis cells and accounts for about 15% of all intracranial neoplasms. They are divided into clinically functioning and non‐functioning. Functioning tumors are characterized by hormonal hypersecretion and related signs and symptoms such as acromegaly due to elevated plasma growth hormone (GH) and/or insulin growth factor 1, amenorrhea–galactorrhea or hypogonadism due to hyperprolactinemia, or Cushing's disease due to hypercortisolism. Non‐functioning tumors do not cause signs and symptoms of hypersecretion, except for hyperprolactinemia due to hypothalamic disconnection.^120^

Generally, the normal pituitary, as well as PitNET tissues, express all five SSTRs; however, it has been demonstrated that the expression of the different SSTR subtypes varies according to the subtype of PitNET.^121^, ^122^, ^123^ Specifically, GH‐ and TSH‐secreting PitNETs express SSTR2 in 90% of cases and SSTR5 to a lesser extent. Conversely, the predominant subtype in ACTH‐secreting PitNETs is SSTR5; however, expression of SSTR2 is also present. Gonadotroph tumors mainly express SSTR3 while in prolactinomas, SSTR1 and SSTR5 are the most representative subtypes.^5^, ^121^, ^124^ Despite the wide evidence of SSTRs expression in PitNETs, PRRT has been rarely used in the management of patients with aggressive or metastatic PitNETs. In fact, to date, only clinical case reports have been published. In 2023, Marques P. reported that of the 27 published cases with available information on response, PRRT resulted in 5 (18%) patients with PR, 8 (30%) demonstrated SD, and 14 patients (52%) had PD.^125^ Notably, some responses to PRRT are remarkable and long‐lasting. However, more than 50% of the published cases had PD after PRRT, probably due to the few PRRT cycles (one or two) that are likely insufficient to stabilize the disease or induce an objective response.^126^

### 3.8 Merkel cell carcinoma

Merkel cell carcinoma (MCC) is a rare, aggressive malignancy of the skin, also known as NE carcinoma of the skin, with high rates of recurrence and distant metastasis.^127^ It was reported that at least one third of MCCs express high levels of SSTRs,^128^ thus allowing for SSA‐based treatments. Experience on the use of PRRT is limited to case reports only. In a recent systematic review, a total of 28 patients from different centers treated with PRRT were collected and analyzed. Radiologic response was available for 19 of 28 patients who received PRRT alone. Six (31.6%) of 19 patients showed objective responses, from partial to complete, and no severe adverse events were reported. The mOS from the start of PRRT was 5 and 8 months in patients receiving PRRT alone or in combination with other active treatments, respectively. mOS from diagnosis was 22 months.^129^

### 3.9 Miscellaneous

**FIGURE 4:**
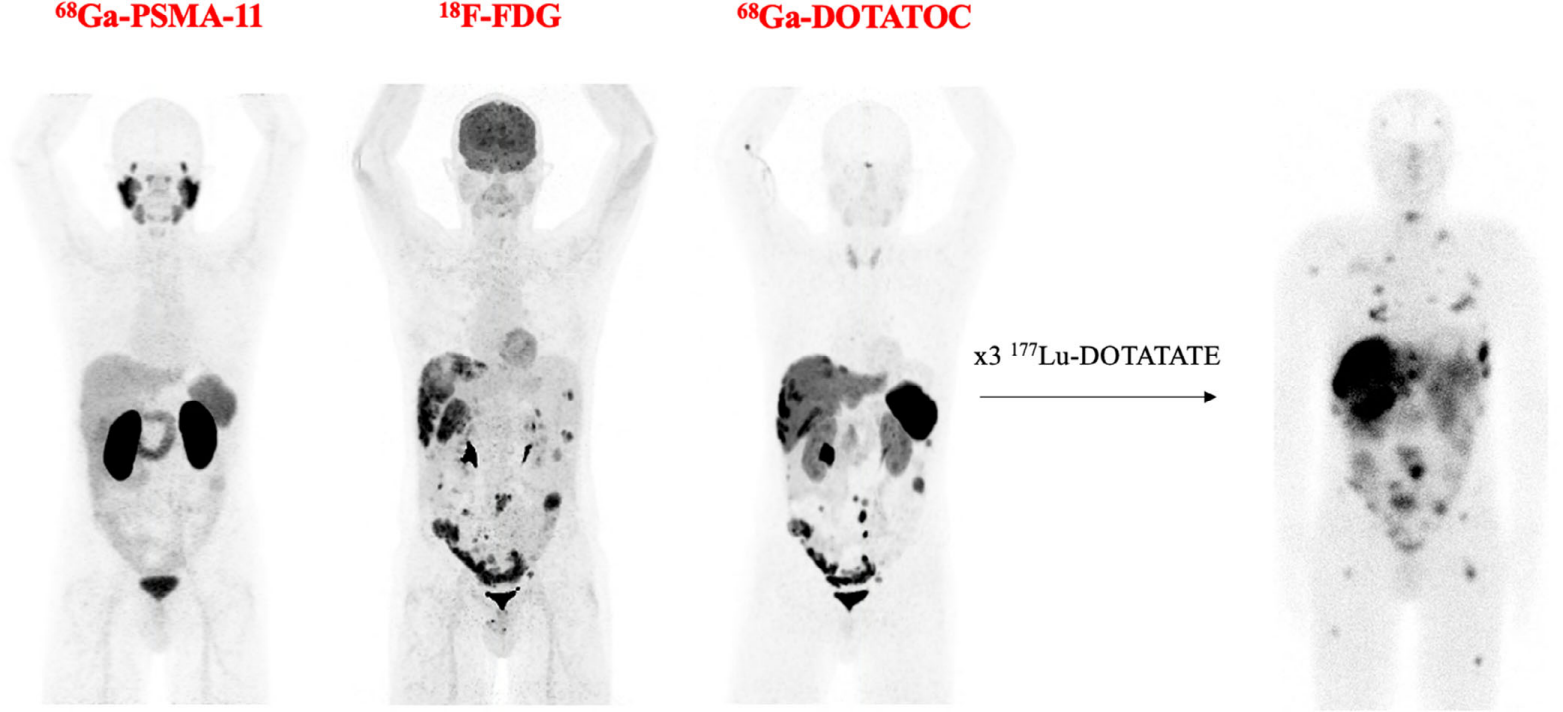
Multi‐tracer imaging with ^68^Ga‐PSMA‐11, ^18^F‐FDG, and ^68^Ga‐DOTATOC in a patient with prostate cancer. The patient was diagnosed with metastatic primary neuroendocrine prostate cancer in 2020 (PSMA PET‐negative, FDG PET‐positive, SSTR PET‐positive). After progression under taxane, the patient was treated with 3 cycles of ^177^Lu‐DOTATATE (cumulated activity 22.8 GBq). The patient progressed during PRRT and died 4 months after the end of treatment. PET, positron emission tomography; PRRT, peptide receptor radionuclide therapy.

As Reubi et al. demonstrated more than 20 years ago,^5^ the expression of SSTR is reported by many different cancer types. Thus, PRRT was used in several tumors with confirmation of SSTR expression, including thymic carcinoid, breast cancer, prostate cancer (Figure 4), colon–rectal cancer, and glioblastoma.^39^, ^130^, ^131^, ^132^, ^133^, ^134^ There are ongoing clinical trials in the recruitment phase that include all SSTR‐positive tumors without distinction of origin (NCT061271, NCT06045260).

## 4 ADVANCEMENTS AND PERSPECTIVES

### 4.1 Combination therapies

Combination treatments have recently gained interest as alternative treatment approaches to enhance PRRT response. Combination treatments have a synergistic effect and potentially upregulate SSTR expression also in non‐GEPNET tumors.^135^, ^136^ Radiosensitizing low‐dose chemotherapy may enhance its effects by inhibiting DNA repair, arresting cell proliferation, increasing DNA damage, or inducing apoptosis. Notably, radiosensitivity varies throughout the cell cycle; it is highest during mitosis and lowest during the S‐phase. Chemotherapeutic agents can arrest cells in mitosis, thereby acting as potent radiation sensitizers.^137^, ^138^

The efficacy of concomitant ^177^Lu‐DOTATATE and capecitabine was evaluated by Yadav et al. in 25 malignant PGLs. Specifically, capecitabine (1250 mg/m^2^) was prescribed for 15 consecutive days commencing on the date of ^177^Lu‐DOTATATE therapy. Twenty‐one of the 24 patients showed a decrease in the CgA level; however, only 7 patients showed a significant decrease in the CgA levels >50%. According to RECIST 1.1, PR was observed in 7 patients (28%), SD in 14 patients (56%), reaching a DCR of about 84%. In the group with PR, the Karnofsky Performance Status showed nearly significant improvement from 62.5 ± 17 to 80 ± 8 (*p* < 0.068), as well as in patients with SD, where a significant improvement in the Karnofsky Performance Status from 66.9 ± 10.4 to 78.5 ± 9.4 (*p* < 0.002) was shown. The predicted mPFS based on RECIST 1.1 criteria was 32 months, and the mOS in the total population was not reached. Considering toxicity profiles, three patients developed Grade 1 lymphocytopenia with no other hematological, renal, or hepatic adverse events reported,^139^ demonstrating no additional toxicity compared to PRRT alone. The safety and efficacy of ^177^Lu‐DOTATATE in combination with carboplatin, etoposide, and atezolizumab vs. chemotherapy alone are under investigation in a Phase I/II clinical trial (NCT05142696) on newly diagnosed extensive‐stage SCLC (ES‐SCLC).

In addition to chemotherapy, the combination of PRRT with immune checkpoint inhibitors has also been explored. The PD‐1/PD‐L1 expression in NETs is a matter of debate. It was suggested that PD‐L1 expression increases with tumor grade in both tumor cells and immune‐infiltrating cells, indicating a complex interaction between tumor aggressiveness and mechanisms of immune escape.^140^ A study conducted by Rösner et al. revealed that PD‐L1 expression in lung NENs is positively correlated with tumor grade, higher Ki‐67 index, and enhanced CXCR4 expression. In contrast, it showed an inverse association with SSTR1 and chromogranin, suggesting that PD‐L1 might be linked to inferior responses to PRRT.^141^ In a preliminary Phase I study on nine extensive‐stage small cell lung cancer (ES‐SCLC) patients treated with PRRT plus Nivolumab, following a standard 3 + 3 design, two activity levels were assessed (Dose level 1: Lutathera 3.7 GBq every 8 weeks for four administrations with nivolumab 240 mg every 2 weeks; activity level 2: Lutathera 7.4 GBq every 8 weeks for four administrations with nivolumab 240 mg every 2 weeks). The authors showed no limiting toxicities at activity level 1, while at activity level 2, one patient developed Grade 3 rash.^142^ Of note, at the time of writing, two trials (i.e., NCT04261855 and NCT05583708) for the evaluation of combined PRRT with immunotherapy (Avelumab and Pembrolizumab, respectively) in patients with MCC are still recruiting.

Inhibiting the DNA repair mechanisms that counteract radiation‐induced damage from PRRT could be an innovative treatment approach. The DNA damage consists of double‐ and/or single‐strand breaks that are repaired by poly (ADP‐ribose) polymerase‐1 (PARP1).^143^ Hence, inhibitors of PARP1 (PARP1i) have become important tools, especially in BRCA1/2mut patients. Promising preclinical data have been published on the combination of ^177^Lu‐DOTATOC with PARP1i in SCLC,^144^ but clinical translation is still awaited.

Future treatment options may also include combining PRRT with EBRT. In 2020, Hartrampf et al. provided long‐term data on a cohort of 10 patients with unresectable meningioma (6 × WHO Grade I, 2 × WHO Grade II, 2 × WHO grading not available) treated with 1 cycle of PRRT followed by EBRT, originally published by Kreiss et al. in 2012.^93^ For the single cycle of PRRT, a mean activity of 7.4 ± 0.3 GBq of ^177^Lu‐DOTATOC/−TATE (DOTATOC, *n* = 6; DOTATATE, *n* = 4) was intravenously injected, resulting in highly heterogeneous tumor doses between 0.2 and 30.7 Gy (median, 7.2 Gy). A median dose of 53.0 Gy (range, 41.8–60.0 Gy) was administered by EBRT. After follow‐up of more than 8 years, no relevant chronic side effects or adverse events >CTC Grade 2 were reported. The combination of PRRT and EBRT resulted in disease stabilization in 7 of the 10 patients. The mPFS was 91.1 months (range, 13.8–111.4) for the entire cohort, with 107.7 months (range, 47.2–111.4) for the patients with controlled disease and 26.2 months (range, 13.8–75.9) for the patients with meningioma progression. The mOS was 105.0 months (range, 38.2–111.4).^94^ In this scenario, the combination of various radiopharmaceuticals, such as ^131^I‐MIBG and ^177^Lu‐DOTATATE, can be used to target simultaneously different pathways in selected tumors.^145^

### 4.2 New alpha isotopes

Alpha‐labeled SSTR‐based analogs have recently gained interest also in NETs. In general, alpha particles generate a high ionization density, resulting in greater double‐strand DNA damage compared to beta particles, which are largely independent of the oxygen levels.^146^, ^147^, ^148^, ^149^, ^150^

The application of TAT, which has been tested preclinically in lung NE models^151^, ^152^ and clinically in patients with NENs of various origins,^153^ could offer an alternative approach to enhance PRRT efficacy. In 2022, a pilot study on nine advanced‐stage PGLs treated with ^225^Ac‐DOTATATE was published: six patients presented with parasympathetic, and three had sympathetic PGL. The mean cumulative activity injected was 42.4 ± 27 (15.54–86.6) MBq, and the median number of cycles administered was 3 (range, 2–9). Among the seven/nine patients evaluated by RECIST 1.1, the best response was PR in four (50%) and SD in three (37.5%) patients. One patient with sympathetic PGL also refractory to the previous ^177^Lu‐PRRT experienced morphological disease progression. The Karnofsky Performance Status showed remarkable improvement in patients with partial tumor regression (60 ± 7 to 85 ± 5, *p* = 0.0050). The intake of analgesics significantly decreased (*p* = 0.0068). No side effects were observed due to concomitant capecitabine treatment. Pre‐existing Grades 1 and 2 anemia was present in four and three patients, respectively, but no worsening was noted during the course of treatment. No Grade 3/4 hematological, kidney, and liver toxicities were reported.^154^

TAT has also been tested in clinical trials. The phase I trial (NCT03466216) of a ^212^Pb‐DOTAMTATE compound called Alphamedix, which was completed in 2021, showed no dose‐limiting toxicity in a group of NEN patients with various primary sites (including lung carcinoid), so a Phase II trial (NCT05153772) of this compound in PRRT‐naive patients is ongoing. Further Phase I/IIa dose escalation studies (NCT05636618; NCT06427798; NCT06479811) of ^212^Pb‐VMT‐α‐NET have expanded the indication to all advanced SSTR2‐positive NETs. In addition, several Phase I studies are evaluating ^225^Ac‐DOTATATE in patients with advanced, SSTR+, well‐differentiated GEP‐NETs who are PRRT‐naïve (NCT06732505) or in progression after ^177^Lu‐based treatment (NCT06732505; NCT05477576). In the past, combinations of ^90^Y‐ and ^177^Lu‐labeled analogs have been studied, but recent advances may shift toward the integration of alpha‐emitting isotopes due to their higher energy transfer and reduced penetration range, which may improve treatment outcomes.

In this context, it is also important to mention the limitations associated with TAT. Firstly, problems of availability and production, half‐life, cost, and the ability to incorporate them chemically and stably into a suitable vector have limited the number of alpha‐radionuclides available for potential clinical use.^155^ In addition, the lack of dosimetry studies, both in vitro and in vivo, in most cases has led to uncertainty in the calculation of the tumor absorbed dose, a key element for further therapy implementation.^156^ Impressive preclinical responses suggest that TAT has a high potential for anti‐tumor efficacy in SSTR2‐overexpressing cancers. However, it is well known that NEN patients often have heterogeneous positive receptor expression. Given the short range of the alpha‐particle, cells with lower receptor expression than neighboring high‐expressing cells are less likely to be irradiated due to the limited crossfire effect.^157^

### 4.3 New pathways

New treatment strategies are emerging for the treatment of NENs based on different targeted receptors. The LUMED trial evaluated the CCK2R antagonist ^177^Lu‐PP‐F11N. In the phase0 study on advanced MTC, the authors first demonstrated that ^177^Lu‐PP‐F11N median absorbed doses for tumors, stomach, kidneys, and bone marrow were 0.88 (interquartile range [IQR], 0.85–1.04), 0.42 (IQR, 0.25–1.01), 0.11 (IQR, 0.07–0.13), and 0.028 (IQR, 0.026–0.034) Gy/GBq, respectively. The median tumor‐to‐stomach dose ratio was 3.34 (IQR, 1.14–4.70), confirming the stomach as the dose‐limiting organ. Adverse reactions (mainly hypotension, flushing, and hypokalemia) were self‐limiting and not higher than Grade 1.^158^ In the context of meningiomas, the PROMENADE Phase 0 study proved the therapeutic efficacy of ^177^Lu‐DOTA‐JR11 in seven progressive treatment‐refractory meningioma patients. Among the total cohort, six patients received one cycle of ^177^Lu‐DOTATOC at an activity of 6.9–7.3 GBq followed by one cycle of ^177^Lu‐DOTA‐JR11 at an activity of 3.3–4.9 GBq. Afterwards, additional ^177^Lu‐DOTA‐JR11 treatment cycles were performed according to clinical needs. The authors observed a median tumor‐to‐bone marrow absorbed dose ratio of 1.4 (range, 0.9–1.9) times higher with ^177^Lu‐DOTA‐JR11. Only 1 of 6 patients showed a slightly lower tumor‐to‐bone marrow absorbed dose ratio with ^177^Lu‐DOTAJR11 than with ^177^Lu‐DOTATOC. In correlation with the dosimetry results, quantitative posttreatment SPECT scans showed more pronounced accumulation in meningioma lesions and in the bone marrow with ^177^Lu‐DOTA‐JR11 than with ^177^Lu‐DOTATOC. Because of the favorable dosimetry results for the SST2 antagonist, one to two additional treatment cycles were performed with ^177^Lu‐DOTA‐JR11, resulting in a DCR of 83% (95% CI, 53%–100%) at least 12 months after inclusion. In all patients, the reported adverse events resolved after a few weeks and there were no Grade 4 or 5 adverse events. Up to 13 months after the first therapy cycle with ^177^Lu‐DOTA‐JR11, there was no worsening of kidney function and no evidence for myelodysplastic syndrome or other neoplasms.^159^ In this scenario, also the NeoRay study (NCT03872778, http://clinicaltrials.gov/show/NCT03872778), a Phase I/IIa Open‐label, Multi‐center Study is ongoing to evaluate the safety, tolerability, and dosimetry of ^177^Lu‐NeoB in patients with advanced solid tumors known to overexpress GRPR, including NENs.

In addition to the new treatment options mentioned above, the use of different routes of administration (locoregional vs. systemic) or their combination (e.g., PRRT plus locoregional treatment for liver metastases NCT04544098), as well as the possibility of using PRRT as an alternative strategy for downstaging tumors (e.g., neoadjuvant treatment before surgery, NCT04609592) is currently being investigated in clinical trials and may be promising approaches to improve the outcome of PRRT also in tumors beyond GEPNET.

## 5 CONCLUSIONS

The present review was designed to collect data on the use of PRRT with SSAs in tumors beyond GEPNET. Although limited by the heterogeneity and paucity of the available data, the present paper shows that many SSTR‐expressing tumors have been treated with PRRT.

In certain types of tumors, such as lung carcinoids, PGLs, and meningiomas, high rates of disease control (up to 80%) have been achieved. Given the limited therapeutic alternatives available for advanced or metastatic stages of these diseases, there is a clear need for results from randomized trials to formally approve PRRT with SSAs for patients who could benefit from this treatment. For other less extensively studied SSTR‐expressing tumors, data are insufficient to draw clear conclusions. In this context, PRRT treatment regimens could be significantly optimized through dosimetry, as empirical fixed‐activity administrations may lead to significant undertreatment.

In the currently evolving scenario, the combination of PRRT with other treatments (e.g., chemotherapy, radiotherapy, immunotherapy), the transition to alpha emitters, as well as the use of alternative approaches (e.g., non‐SSTR‐based radionuclide treatment, different routes of administration) may also find a way in NENs beyond GEPNET. Results of ongoing clinical trials are awaited to confirm the efficacy of these innovative approaches and hopefully pave the way to new avenues for thera(g)nostic.

## AUTHOR CONTRIBUTIONS

**Giulia Santo:** Conceptualization; data curation; writing – original draft. **Gianpaolo di Santo:** Visualization; writing – review and editing. **Francesco Cicone:** Visualization; writing – review and editing. **Irene Virgolini:** Writing – review and editing; supervision; conceptualization.

## CONFLICT OF INTEREST STATEMENT

The authors declare no conflicts of interest.

### PEER REVIEW

The peer review history for this article is available at https://www.webofscience.com/api/gateway/wos/peer-review/10.1111/jne.70013.

## PATIENT CONSENT

Informed consent for the publication of images was received from all patients who appear in the manuscript.

## Data Availability

Data sharing not applicable to this article as no datasets were generated or analysed during the current study.

## References

[1] Bodei L , Mueller‐Brand J , Baum RP , et al. The joint IAEA, EANM, and SNMMI practical guidance on peptide receptor radionuclide therapy (PRRNT) in neuroendocrine tumours. Eur J Nucl Med Mol Imaging. 2013;40(5):800‐816. doi:10.1007/s00259-012-2330-6 23389427 PMC3622744

[2] Brazeau P , Vale W , Burgus R , et al. Hypothalamic polypeptide that inhibits the secretion of immunoreactive pituitary growth hormone. Science. 1973;179(4068):77‐79. doi:10.1126/science.179.4068.77 4682131

[3] Van Op den Bosch J , Adriaensen D , Van Nassauw L , Timmermans JP . The role(s) of somatostatin, structurally related peptides and somatostatin receptors in the gastrointestinal tract: a review. Regul Pept. 2009;156(1–3):1‐8. doi:10.1016/j.regpep.2009.04.003 19362110

[4] Theodoropoulou M , Stalla GK . Somatostatin receptors: from signaling to clinical practice. Front Neuroendocrinol. 2013;34(3):228‐252. doi:10.1016/j.yfrne.2013.07.005 23872332

[5] Reubi JC , Waser B , Schaer JC , Laissue JA . Somatostatin receptor sst1‐sst5 expression in normal and neoplastic human tissues using receptor autoradiography with subtype‐selective ligands. Eur J Nucl Med. 2001;28(7):836‐846. doi:10.1007/s002590100541 11504080

[6] Strosberg J , El‐Haddad G , Wolin E , et al. Phase 3 trial of ^177^Lu‐dotatate for midgut neuroendocrine tumors. N Engl J Med. 2017;376(2):125‐135. doi:10.1056/NEJMoa1607427 28076709 PMC5895095

[7] Virgolini I , Britton K , Buscombe J , Moncayo R , Paganelli G , Riva P . In‐ and Y‐DOTA‐lanreotide: results and implications of the MAURITIUS trial. Semin Nucl Med. 2002;32(2):148‐155. doi:10.1053/snuc.2002.31565 11965610

[8] Evangelou G , Vamvakaris I , Papafili A , Anagnostakis M , Peppa M . Lung NETs and GEPNETs: one cancer with different origins or two distinct cancers? Cancers Basel. 2024;16(6):1177. doi:10.3390/cancers16061177 38539512 PMC10969423

[9] Bodei L , Schöder H , Baum RP , et al. Molecular profiling of neuroendocrine tumours to predict response and toxicity to peptide receptor radionuclide therapy. Lancet Oncol. 2020;21(9):e431‐e443. doi:10.1016/S1470-2045(20)30323-5 32888472 PMC8385643

[10] Bischoff P , Trinks A , Wiederspahn J , et al. The single‐cell transcriptional landscape of lung carcinoid tumors. Int J Cancer. 2022;150(12):2058‐2071. doi:10.1002/ijc.33995 35262195

[11] Chmiel P , Rychcik‐Pazyrska P , Stec R . Defining tumor microenvironment as a possible target for effective GEP‐NENs immunotherapy—a systematic review. Cancers Basel. 2023;15(21):5232. doi:10.3390/cancers15215232 37958406 PMC10648089

[12] Shi C , Morse MA . Mechanisms of resistance in gastroenteropancreatic neuroendocrine tumors. Cancers Basel. 2022;14(24):6114. doi:10.3390/cancers14246114 36551599 PMC9776394

[13] Parliament MB , Murray D . Single nucleotide polymorphisms of DNA repair genes as predictors of radioresponse. Semin Radiat Oncol. 2010;20(4):232‐240. doi:10.1016/j.semradonc.2010.05.003 20832015

[14] Deppen SA , Blume J , Bobbey AJ , et al. 68Ga‐DOTATATE compared with 111In‐DTPA‐octreotide and conventional imaging for pulmonary and Gastroenteropancreatic neuroendocrine tumors: a systematic review and meta‐analysis. J Nucl Med. 2016;57(6):872‐878. doi:10.2967/jnumed.115.165803 26769864 PMC5362941

[15] Pauwels E , Cleeren F , Bormans G , Deroose CM . Somatostatin receptor PET ligands—the next generation for clinical practice. Am J Nucl Med Mol Imaging. 2018;8(5):311‐331. 30510849 PMC6261874

[16] Bozkurt MF , Virgolini I , Balogova S , et al. Guideline for PET/CT imaging of neuroendocrine neoplasms with 68Ga‐DOTA‐conjugated somatostatin receptor targeting peptides and 18F‐DOPA. Eur J Nucl Med Mol Imaging. 2017;44(9):1588‐1601. doi:10.1007/s00259-017-3728-y 28547177

[17] Pfeifer A , Knigge U , Mortensen J , et al. Clinical PET of neuroendocrine tumors using 64CuDOTATATE: first‐in‐humans study. J Nucl Med. 2012;53:1207‐1215. 22782315 10.2967/jnumed.111.101469

[18] Santo G , Di Santo G , Virgolini I . Peptide receptor radionuclide therapy of neuroendocrine tumors: agonist, antagonist and alternatives. Semin Nucl Med. 2024;54(4):557‐569. doi:10.1053/j.semnuclmed.2024.02.002 38490913

[19] Wild D , Fani M , Fischer R , et al. Comparison of somatostatin receptor agonist and antagonist for peptide receptor radionuclide therapy: a pilot study. J Nucl Med. 2014;55:1248‐1252. 24963127 10.2967/jnumed.114.138834

[20] Wild D , Antwi K , Fani M , Christ ER . Glucagon‐like peptide‐1 receptor as emerging target: will it make it to the clinic? J Nucl Med. 2021;62(suppl. 2):44S‐50S. doi:10.2967/jnumed.120.246009 34230073

[21] Lapa C , Lückerath K , Rudelius M , et al. [68Ga]Pentixafor‐PET/CT for imaging of chemokine receptor 4 expression in small cell lung cancer. Oncotarget. 2016;7(8):9288‐9295. doi:10.18632/oncotarget.7063 26843617 PMC4891040

[22] von Guggenberg E , Uprimny C , Klinger M , et al. Preliminary clinical experience with cholecystokinin‐2 receptor PET/CT using the 68Ga‐labeled minigastrin analog DOTA‐MGS5 in patients with medullary thyroid cancer. J Nucl Med. 2023;64(6):859‐862. doi:10.2967/jnumed.122.264977 36657979

[23] Di Santo G , Santo G , Martinovic V , et al. Cholecystokinin‐2 receptor targeting by [68Ga]Ga‐DOTA‐MGS5 PET/CT in a patient with extensive disease small cell lung cancer. Eur J Nucl Med Mol Imaging. 2024;51(9):2848‐2849. doi:10.1007/s00259-024-06749-z 38740575 PMC11224066

[24] Moreno P , Ramos‐Álvarez I , Moody TW , Jensen RT . Bombesin related peptides/receptors and their promising therapeutic roles in cancer imaging, targeting and treatment. Expert Opin Ther Targets. 2016;20(9):1055‐1073. doi:10.1517/14728222.2016.1164694 26981612 PMC5067074

[25] Ito T , Jensen RT . Molecular imaging in neuroendocrine tumors: recent advances, controversies, unresolved issues, and roles in management. Curr Opin Endocrinol Diabetes Obes. 2017;24(1):15‐24. doi:10.1097/MED.0000000000000300 27875420 PMC5195891

[26] Krenning EP , Kooij PP , Bakker WH , et al. Radiotherapy with a radiolabeled somatostatin analogue, [111In‐DTPA‐D‐Phe1]‐octreotide. A case history. Ann N Y Acad Sci. 1994;733:496‐506. doi:10.1111/j.1749-6632.1994.tb17300.x 7978900

[27] Targeted Alpha Therapy Working Group , Parker C , Lewington V , et al. Targeted alpha therapy, an emerging class of cancer agents: a review. JAMA Oncol. 2018;4(12):1765‐1772. doi:10.1001/jamaoncol.2018.4044 30326033

[28] Mariniello A , Bodei L , Tinelli C , et al. Long‐term results of PRRT in advanced bronchopulmonary carcinoid. Eur J Nucl Med Mol Imaging. 2016;43(3):441‐452. doi:10.1007/s00259-015-3190-7 26392198

[29] Ianniello A , Sansovini M , Severi S , et al. Peptide receptor radionuclide therapy with (177)Lu‐DOTATATE in advanced bronchial carcinoids: prognostic role of thyroid transcription factor 1 and (18)F‐FDG PET. Eur J Nucl Med Mol Imaging. 2016;43(6):1040‐1046. doi:10.1007/s00259-015-3262-8 26611427

[30] Sabet A , Haug AR , Eiden C , et al. Efficacy of peptide receptor radionuclide therapy with ^177^Lu‐octreotate in metastatic pulmonary neuroendocrine tumors: a dual‐centre analysis. Am J Nucl Med Mol Imaging. 2017;7(2):74‐83. 28533939 PMC5435613

[31] Mirvis E , Toumpanakis C , Mandair D , Gnanasegaran G , Caplin M , Navalkissoor S . Efficacy and tolerability of peptide receptor radionuclide therapy (PRRT) in advanced metastatic bronchial neuroendocrine tumours (NETs). Lung Cancer. 2020;150:70‐75. doi:10.1016/j.lungcan.2020.10.005 33075738

[32] Minutoli F , Cardile D , Laudicella R , Vento A , Pagano B , Baldari S . Peptide receptor radionuclide therapy of pulmonary neuroendocrine neoplasms: a single‐Centre experience. Nucl Med Mol Imaging. 2021;55(1):38‐45. doi:10.1007/s13139-020-00679-y 33643488 PMC7881077

[33] Parghane RV , Talole S , Prabhash K , Basu S . Clinical response profile of metastatic/advanced pulmonary neuroendocrine tumors to peptide receptor radionuclide therapy with ^177^Lu‐DOTATATE. Clin Nucl Med. 2017;42(6):428‐435. doi:10.1097/RLU.0000000000001639 28319500

[34] Lim LE , Chan DL , Thomas D , et al. Australian experience of peptide receptor radionuclide therapy in lung neuroendocrine tumours. Oncotarget. 2020;11(27):2636‐2646. Published 2020 Jul 7. doi:10.18632/oncotarget.27659 32676165 PMC7343632

[35] Zidan L , Iravani A , Oleinikov K , et al. Efficacy and safety of ^177^Lu‐DOTATATE in lung neuroendocrine tumors: a bicenter study. J Nucl Med. 2022;63(2):218‐225. doi:10.2967/jnumed.120.260760 34049983 PMC8805789

[36] Thoracic Tumours . WHO Classification of Tumours. Vol 5. 5th ed. WHO Classification of Tumours Editorial Board; 2021.

[37] Vocino Trucco G , Righi L , Volante M , Papotti M . Updates on lung neuroendocrine neoplasm classification. Histopathology. 2024;84(1):67‐85. doi:10.1111/his.15058 37794655

[38] Kanakis G , Grimelius L , Spathis A , et al. Expression of somatostatin receptors 1–5 and dopamine receptor 2 in lung carcinoids: implications for a therapeutic role. Neuroendocrinology. 2015;101(3):211‐222. doi:10.1159/000381061 25765100

[39] Baudin E , Caplin M , Garcia‐Carbonero R , et al. Lung and thymic carcinoids: ESMO Clinical Practice Guidelines for diagnosis, treatment and follow‐up. Ann Oncol. 2021;32(11):1453‐1455. doi:10.1016/j.annonc.2021.08.2150 34598840

[40] Yao JC , Fazio N , Singh S , et al. Everolimus for the treatment of advanced, non‐functional neuroendocrine tumours of the lung or gastrointestinal tract (RADIANT‐4): a randomised, placebo‐controlled, phase 3 study. Lancet. 2016;387(10022):968‐977. doi:10.1016/S0140-6736(15)00817-X 26703889 PMC6063317

[41] Kwekkeboom DJ , de Herder WW , Kam BL , et al. Treatment with the radiolabeled somatostatin analog [177 Lu‐DOTA 0,Tyr3]octreotate: toxicity, efficacy, and survival. J Clin Oncol. 2008;26(13):2124‐2130. doi:10.1200/JCO.2007.15.2553 18445841

[42] Jayaprakasam VS , Bodei L . Neuroendocrine tumor therapy response assessment. PET Clin. 2023;18(2):267‐286. doi:10.1016/j.cpet.2022.11.009 36858748

[43] Forrer F , Riedweg I , Maecke HR , Mueller‐Brand J . Radiolabeled DOTATOC in patients with advanced paraganglioma and pheochromocytoma. Q J Nucl Med Mol Imaging. 2008;52(4):334‐340. 18480742

[44] Nastos K , Cheung VTF , Toumpanakis C , et al. Peptide receptor radionuclide treatment and (^131^)I‐MIBG in the management of patients with metastatic/progressive phaeochromocytomas and paragangliomas. J Surg Oncol. 2017;115(4):425‐434. doi:10.1002/jso.24553 28166370

[45] Kong G , Grozinsky‐Glasberg S , Hofman MS , et al. Efficacy of peptide receptor radionuclide therapy for functional metastatic paraganglioma and pheochromocytoma. J Clin Endocrinol Metab. 2017;102(9):3278‐3287. doi:10.1210/jc.2017-00816 28605448

[46] Zandee WT , Feelders RA , Smit Duijzentkunst DA , et al. Treatment of inoperable or metastatic paragangliomas and pheochromocytomas with peptide receptor radionuclide therapy using ^177^Lu‐DOTATATE. Eur J Endocrinol. 2019;181(1):45‐53. doi:10.1530/EJE-18-0901 31067510

[47] Vyakaranam AR , Crona J , Norlén O , et al. Favorable outcome in patients with pheochromocytoma and paraganglioma treated with ^177^Lu‐DOTATATE. Cancers Basel. 2019;11(7):909. doi:10.3390/cancers11070909 31261748 PMC6678507

[48] Kolasinska‐Ćwikła A , Pęczkowska M , Ćwikła JB , et al. A clinical efficacy of PRRT in patients with advanced, nonresectable, paraganglioma–pheochromocytoma, related to SDHx gene mutation. J Clin Med. 2019;8(7):952. doi:10.3390/jcm8070952 31262070 PMC6678858

[49] Jaiswal SK , Sarathi V , Memon SS , et al. ^177^Lu‐DOTATATE therapy in metastatic/inoperable pheochromocytoma–paraganglioma. Endocr Connect. 2020;9(9):864‐873. doi:10.1530/EC-20-0292 32784267 PMC7487189

[50] Severi S , Bongiovanni A , Ferrara M , et al. Peptide receptor radionuclide therapy in patients with metastatic progressive pheochromocytoma and paraganglioma: long‐term toxicity, efficacy and prognostic biomarker data of phase II clinical trials. ESMO Open. 2021;6(4):100171. doi:10.1016/j.esmoop.2021.100171 34139487 PMC8219772

[51] Nilica B , Svirydenka H , Mair C , Stephan Kroiss A , Gabriel M , Virgolini I . Personalized thera(g)nostic approach in patients with paraganglioma: peptide receptor radionuclide therapy. Clin Oncol. 2021;6:1824.

[52] Prado‐Wohlwend S , Del Olmo‐García MI , Bello‐Arques P , Merino‐Torres JF . [^177^Lu]Lu‐DOTA‐TATE and [^131^I]MIBG phenotypic imaging‐based therapy in metastatic/inoperable pheochromocytomas and paragangliomas: comparative results in a single center. Front Endocrinol Lausanne. 2022;13:778322. doi:10.3389/fendo.2022.778322 35197929 PMC8859101

[53] Rubino M , Di Stasio GD , Bodei L , et al. Peptide receptor radionuclide therapy with ^177^Lu‐ or 90Y‐SSTR peptides in malignant pheochromocytomas (PCCs) and paragangliomas (PGLs): results from a single institutional retrospective analysis. Endocrine. 2024;84(2):704‐710. doi:10.1007/s12020-024-03707-5 38324106 PMC12054632

[54] Mete O , Asa SL , Gill AJ , Kimura N , de Krijger RR , Tischler A . Overview of the 2022 WHO classification of paragangliomas and pheochromocytomas. Endocr Pathol. 2022;33(1):90‐114. doi:10.1007/s12022-022-09704-6 35285002

[55] Flynn A , Benn D , Clifton‐Bligh R , et al. The genomic landscape of phaeochromocytoma. J Pathol. 2015;236(1):78‐89. doi:10.1002/path.4503 25545346

[56] Neumann HP , Pawlu C , Peczkowska M , et al. Distinct clinical features of paraganglioma syndromes associated with SDHB and SDHD gene mutations. JAMA. 2004;292(8):943‐951. doi:10.1001/jama.292.8.943 15328326

[57] Pasini B , McWhinney SR , Bei T , et al. Clinical and molecular genetics of patients with the Carney–Stratakis syndrome and germline mutations of the genes coding for the succinate dehydrogenase subunits SDHB, SDHC, and SDHD. Eur J Hum Genet. 2008;16(1):79‐88. doi:10.1038/sj.ejhg.5201904 17667967

[58] Fassnacht M , Assie G , Baudin E , et al. Adrenocortical carcinomas and malignant phaeochromocytomas: ESMO‐EURACAN Clinical Practice Guidelines for diagnosis, treatment and follow‐up. Ann Oncol. 2020;31(11):1476‐1490. doi:10.1016/j.annonc.2020.08.2099 32861807

[59] Gonias S , Goldsby R , Matthay KK , et al. Phase II study of high‐dose [^131^I]metaiodobenzylguanidine therapy for patients with metastatic pheochromocytoma and paraganglioma. J Clin Oncol. 2009;27(25):4162‐4168. doi:10.1200/JCO.2008.21.3496 19636009 PMC2734428

[60] Pryma DA , Chin BB , Noto RB , et al. Efficacy and safety of high‐specific‐activity ^131^I‐MIBG therapy in patients with advanced pheochromocytoma or paraganglioma. J Nucl Med. 2019;60(5):623‐630. doi:10.2967/jnumed.118.217463 30291194 PMC6495236

[61] Reubi JC , Waser B , Khosla S , et al. In vitro and in vivo detection of somatostatin receptors in pheochromocytomas and paragangliomas. J Clin Endocrinol Metab. 1992;74(5):1082‐1089. doi:10.1210/jcem.74.5.1349024 1349024

[62] Elston MS , Meyer‐Rochow GY , Conaglen HM , et al. Increased SSTR2A and SSTR3 expression in succinate dehydrogenase‐deficient pheochromocytomas and paragangliomas. Hum Pathol. 2015;46(3):390‐396. doi:10.1016/j.humpath.2014.11.012 25554089

[63] Benn DE , Robinson BG , Clifton‐Bligh RJ . 15 Years of paraganglioma: clinical manifestations of paraganglioma syndromes types 1–5. Endocr Relat Cancer. 2015;22(4):T91‐T103. doi:10.1530/ERC-15-0268 26273102 PMC4532956

[64] Bodei L , Handkiewicz‐Junak D , Grana C , et al. Receptor radionuclide therapy with 90Y‐DOTATOC in patients with medullary thyroid carcinomas. Cancer Biother Radiopharm. 2004;19(1):65‐71. doi:10.1089/108497804773391694 15068613

[65] Iten F , Müller B , Schindler C , et al. Response to [90Yttrium‐DOTA]‐TOC treatment is associated with long‐term survival benefit in metastasized medullary thyroid cancer: a phase II clinical trial. Clin Cancer Res. 2007;13(22 Pt 1):6696‐6702. doi:10.1158/1078-0432.CCR-07-0935 18006770

[66] Beukhof CM , Brabander T , van Nederveen FH , et al. Peptide receptor radionuclide therapy in patients with medullary thyroid carcinoma: predictors and pitfalls. BMC Cancer. 2019;19(1):325. doi:10.1186/s12885-019-5540-5 30953466 PMC6451300

[67] Parghane RV , Naik C , Talole S , et al. Clinical utility of ^177^Lu‐DOTATATE PRRT in somatostatin receptor‐positive metastatic medullary carcinoma of thyroid patients with assessment of efficacy, survival analysis, prognostic variables, and toxicity. Head Neck. 2020;42(3):401‐416. doi:10.1002/hed.26024 31755622

[68] Liu Q , Kulkarni HR , Zhao T , et al. Peptide receptor radionuclide therapy in patients with advanced progressive medullary thyroid cancer: efficacy, safety, and survival predictors. Clin Nucl Med. 2023;48(3):221‐227. doi:10.1097/RLU.0000000000004539 36723881

[69] Wells SA Jr , Asa SL , Dralle H , et al. Revised American Thyroid Association guidelines for the management of medullary thyroid carcinoma. Thyroid. 2015;25(6):567‐610. doi:10.1089/thy.2014.0335 25810047 PMC4490627

[70] Filetti S , Durante C , Hartl D , et al. Thyroid cancer: ESMO Clinical Practice Guidelines for diagnosis, treatment and follow‐up. Ann Oncol. 2019;30(12):1856‐1883. doi:10.1093/annonc/mdz400 31549998

[71] Wells SA Jr , Robinson BG , Gagel RF , et al. Vandetanib in patients with locally advanced or metastatic medullary thyroid cancer: a randomized, double‐blind phase III trial. J Clin Oncol. 2012;30(2):134‐141. doi:10.1200/JCO.2011.35.5040 22025146 PMC3675689

[72] Zatelli MC , Piccin D , Tagliati F , et al. Selective activation of somatostatin receptor subtypes differentially modulates secretion and viability in human medullary thyroid carcinoma primary cultures: potential clinical perspectives. J Clin Endocrinol Metab. 2006;91(6):2218‐2224. doi:10.1210/jc.2006-0334 16569735

[73] Kwekkeboom DJ , Reubi JC , Lamberts SW , et al. In vivo somatostatin receptor imaging in medullary thyroid carcinoma. J Clin Endocrinol Metab. 1993;76(6):1413‐1417. doi:10.1210/jcem.76.6.8501144 8501144

[74] Caplin ME , Mielcarek W , Buscombe JR , et al. Toxicity of high‐activity 111In‐octreotide therapy in patients with disseminated neuroendocrine tumours. Nucl Med Commun. 2000;21(1):97‐102. doi:10.1097/00006231-200001000-00016 10717909

[75] Buscombe JR , Caplin ME , Hilson AJ . Long‐term efficacy of high‐activity 111in‐pentetreotide therapy in patients with disseminated neuroendocrine tumors. J Nucl Med. 2003;44(1):1‐6. 12515868

[76] Lee DY , Kim YI . Peptide receptor radionuclide therapy in patients with differentiated thyroid cancer: a meta‐analysis. Clin Nucl Med. 2020;45(8):604‐610. doi:10.1097/RLU.0000000000003110 32520503

[77] Bartolomei M , Bodei L , De Cicco C , et al. Peptide receptor radionuclide therapy with (90)Y‐DOTATOC in recurrent meningioma. Eur J Nucl Med Mol Imaging. 2009;36(9):1407‐1416. doi:10.1007/s00259-009-1115-z 19319527

[78] Gerster‐Gilliéron K , Forrer F , Maecke H , Mueller‐Brand J , Merlo A , Cordier D . 90Y‐DOTATOC as a therapeutic option for complex recurrent or progressive meningiomas. J Nucl Med. 2015;56(11):1748‐1751. doi:10.2967/jnumed.115.155853 26294303

[79] Marincek N , Radojewski P , Dumont RA , et al. Somatostatin receptor‐targeted radiopeptide therapy with 90Y‐DOTATOC and ^177^Lu‐DOTATOC in progressive meningioma: long‐term results of a phase II clinical trial. J Nucl Med. 2015;56(2):171‐176. doi:10.2967/jnumed.114.147256 25593116

[80] Seystahl K , Stoecklein V , Schüller U , et al. Somatostatin receptor‐targeted radionuclide therapy for progressive meningioma: benefit linked to ^68^Ga‐DOTATATE/‐TOC uptake. Neuro Oncol. 2016;18(11):1538‐1547. doi:10.1093/neuonc/now060 27106404 PMC5063513

[81] Minczeles NS , Bos EM , de Leeuw RC , et al. Efficacy and safety of peptide receptor radionuclide therapy with [^177^Lu]Lu‐DOTA‐TATE in 15 patients with progressive treatment‐refractory meningioma. Eur J Nucl Med Mol Imaging. 2023;50(4):1195‐1204. doi:10.1007/s00259-022-06044-9 36454268

[82] Kurz SC , Zan E , Cordova C , et al. Evaluation of the SSTR2‐targeted radiopharmaceutical ^177^Lu‐DOTATATE and SSTR2‐specific ^68^Ga‐DOTATATE PET as imaging biomarker in patients with intracranial meningioma. Clin Cancer Res. 2024;30(4):680‐686. doi:10.1158/1078-0432.CCR-23-2533 38048045

[83] Gittleman HR , Ostrom QT , Rouse CD , et al. Trends in central nervous system tumor incidence relative to other common cancers in adults, adolescents, and children in the United States, 2000 to 2010. Cancer. 2015;121(1):102‐112. doi:10.1002/cncr.29015 25155924 PMC4298242

[84] Maggio I , Franceschi E , Tosoni A , et al. Meningioma: not always a benign tumor. A review of advances in the treatment of meningiomas. CNS Oncol. 2021;10(2):CNS72. doi:10.2217/cns-2021-0003 34015955 PMC8162186

[85] Torp SH , Solheim O , Skjulsvik AJ . The WHO 2021 classification of central nervous system tumours: a practical update on what neurosurgeons need to know‐a minireview. Acta Neurochir. 2022;164(9):2453‐2464. doi:10.1007/s00701-022-05301-y 35879477 PMC9427889

[86] Goldbrunner R , Stavrinou P , Jenkinson MD , et al. EANO guideline on the diagnosis and management of meningiomas. Neuro Oncol. 2021;23(11):1821‐1834. doi:10.1093/neuonc/noab150 34181733 PMC8563316

[87] Apra C , Peyre M , Kalamarides M . Current treatment options for meningioma. Expert Rev Neurother. 2018;18(3):241‐249. doi:10.1080/14737175.2018.1429920 29338455

[88] Albert NL , Preusser M , Traub‐Weidinger T , et al. Joint EANM/EANO/RANO/SNMMI practice guideline/procedure standards for diagnostics and therapy (theranostics) of meningiomas using radiolabeled somatostatin receptor ligands: version 1.0. Eur J Nucl Med Mol Imaging. 2024;51(12):3662‐3679. doi:10.1007/s00259-024-06783-x 38898354 PMC11445317

[89] Arena S , Barbieri F , Thellung S , et al. Expression of somatostatin receptor mRNA in human meningiomas and their implication in in vitro antiproliferative activity. J Neurooncol. 2004;66(1–2):155‐166. doi:10.1023/b:neon.0000013498.19981.55 15015781

[90] Dutour A , Kumar U , Panetta R , et al. Expression of somatostatin receptor subtypes in human brain tumors. Int J Cancer. 1998;76(5):620‐627. doi:10.1002/(sici)1097-0215(19980529)76:5<620::aid-ijc2>3.0.co;2-s 9610716

[91] Minutoli F , Amato E , Sindoni A , et al. Peptide receptor radionuclide therapy in patients with inoperable meningiomas: our experience and review of the literature. Cancer Biother Radiopharm. 2014;29(5):193‐199. doi:10.1089/cbr.2013.1599 24811687

[92] Mirian C , Duun‐Henriksen AK , Maier A , et al. Somatostatin receptor‐targeted radiopeptide therapy in treatment‐refractory meningioma: individual patient data meta‐analysis. J Nucl Med. 2021;62(4):507‐513. doi:10.2967/jnumed.120.249607 32859705

[93] Kreissl MC , Hänscheid H , Löhr M , et al. Combination of peptide receptor radionuclide therapy with fractionated external beam radiotherapy for treatment of advanced symptomatic meningioma. Radiat Oncol. 2012;7:99. doi:10.1186/1748-717X-7-99 22720902 PMC3439242

[94] Hartrampf PE , Hänscheid H , Kertels O , et al. Long‐term results of multimodal peptide receptor radionuclide therapy and fractionated external beam radiotherapy for treatment of advanced symptomatic meningioma. Clin Transl Radiat Oncol. 2020;22:29‐32. doi:10.1016/j.ctro.2020.03.002 32195377 PMC7075763

[95] Boursier C , Zaragori T , Bros M , et al. Semi‐automated segmentation methods of SSTR PET for dosimetry prediction in refractory meningioma patients treated by SSTR‐targeted peptide receptor radionuclide therapy. Eur Radiol. 2023;33(10):7089‐7098. doi:10.1007/s00330-023-09697-8 37148355

[96] Cicone F , Gnesin S , Santo G , et al. Do we need dosimetry for the optimization of theranostics in CNS tumors? Neuro Oncol. 2024;26(suppl):S242‐S258. doi:10.1093/neuonc/noae200 39351795 PMC11631076

[97] Mair MJ , Tabouret E , Johnson DR , et al. Radioligand therapies in meningioma: evidence and future directions. Neuro Oncol. 2024;26(suppl):S215‐S228. doi:10.1093/neuonc/noae069 38702966 PMC11631075

[98] Huang RY , Bi WL , Weller M , et al. Proposed response assessment and endpoints for meningioma clinical trials: report from the Response Assessment in Neuro‐Oncology Working Group. Neuro Oncol. 2019;21(1):26‐36. doi:10.1093/neuonc/noy137 30137421 PMC6303427

[99] Schlumberger M , Tahara M , Wirth LJ , et al. Lenvatinib versus placebo in radioiodine‐refractory thyroid cancer. N Engl J Med. 2015;372(7):621‐630. doi:10.1056/NEJMoa1406470 25671254

[100] Brose MS , Nutting CM , Jarzab B , et al. Sorafenib in radioactive iodine‐refractory, locally advanced or metastatic differentiated thyroid cancer: a randomised, double‐blind, phase 3 trial. Lancet. 2014;384(9940):319‐328. doi:10.1016/S0140-6736(14)60421-9 24768112 PMC4366116

[101] Reubi JC , Laissue J , Krenning E , Lamberts SW . Somatostatin receptors in human cancer: incidence, characteristics, functional correlates and clinical implications. J Steroid Biochem Mol Biol. 1992;43(1–3):27‐35. doi:10.1016/0960-0760(92)90184-k 1356016

[102] Ain KB , Taylor KD , Tofiq S , Venkataraman G . Somatostatin receptor subtype expression in human thyroid and thyroid carcinoma cell lines. J Clin Endocrinol Metab. 1997;82(6):1857‐1862. doi:10.1210/jcem.82.6.4013 9177396

[103] Klagge A , Krause K , Schierle K , Steinert F , Dralle H , Fuhrer D . Somatostatin receptor subtype expression in human thyroid tumours. Horm Metab Res. 2010;42(4):237‐240. doi:10.1055/s-0029-1243636 20094970

[104] Gabriel M , Froehlich F , Decristoforo C , et al. ^99m^Tc‐EDDA/HYNIC‐TOC and ^18^F‐FDG in thyroid cancer patients with negative ^131^I whole‐body scans. Eur J Nucl Med Mol Imaging. 2004;31(3):330‐341. doi:10.1007/s00259-003-1376-x 14625664

[105] Roll W , Riemann B , Schäfers M , Stegger L , Vrachimis A . ^177^Lu‐DOTATATE therapy in radioiodine‐refractory differentiated thyroid cancer: a single center experience. Clin Nucl Med. 2018;43(10):346‐351. doi:10.1097/RLU.0000000000002219 30059430

[106] Czepczyński R , Matysiak‐Grześ M , Gryczyńska M , et al. Peptide receptor radionuclide therapy of differentiated thyroid cancer: efficacy and toxicity. Arch Immunol Ther Exp (Warsz). 2015;63(2):147‐154. doi:10.1007/s00005-014-0318-6 25403743 PMC4359293

[107] Versari A , Sollini M , Frasoldati A , et al. Differentiated thyroid cancer: a new perspective with radiolabeled somatostatin analogues for imaging and treatment of patients. Thyroid. 2014;24(4):715‐726. doi:10.1089/thy.2013.0225 24102584

[108] Iten F , Muller B , Schindler C , et al. [^90^Yttrium‐DOTA]‐TOC response is associated with survival benefit in iodine‐refractory thyroid cancer: long‐term results of a phase 2 clinical trial. Cancer. 2009;115(10):2052‐2062. doi:10.1002/cncr.24272 19280592

[109] Johnsen JI , Dyberg C , Wickström M . Neuroblastoma‐a neural crest derived embryonal malignancy. Front Mol Neurosci. 2019;12:9. doi:10.3389/fnmol.2019.00009 30760980 PMC6361784

[110] Cheung NK , Dyer MA . Neuroblastoma: developmental biology, cancer genomics and immunotherapy. Nat Rev Cancer. 2013;13(6):397‐411. doi:10.1038/nrc3526 23702928 PMC4386662

[111] Alexander N , Vali R , Ahmadzadehfar H , Shammas A , Baruchel S . Review: the role of radiolabeled DOTA‐conjugated peptides for imaging and treatment of childhood Neuroblastoma. Curr Radiopharm. 2018;11(1):14‐21. doi:10.2174/1874471011666171215093112 29243585

[112] Gains JE , Sebire NJ , Moroz V , Wheatley K , Gaze MN . Immunohistochemical evaluation of molecular radiotherapy target expression in neuroblastoma tissue. Eur J Nucl Med Mol Imaging. 2018;45(3):402‐411. doi:10.1007/s00259-017-3856-4 29043399

[113] Georgantzi K , Tsolakis AV , Stridsberg M , Jakobson A , Christofferson R , Janson ET . Differentiated expression of somatostatin receptor subtypes in experimental models and clinical neuroblastoma. Pediatr Blood Cancer. 2011;56(4):584‐589. doi:10.1002/pbc.22913 21298743

[114] Alexander N , Marrano P , Thorner P , et al. Prevalence and clinical correlations of somatostatin receptor‐2 (SSTR2) expression in Neuroblastoma. J Pediatr Hematol Oncol. 2019;41(3):222‐227. doi:10.1097/MPH.0000000000001326 30334904 PMC6600817

[115] Gains JE , Bomanji JB , Fersht NL , et al. ^177^Lu‐DOTATATE molecular radiotherapy for childhood neuroblastoma. J Nucl Med. 2011;52(7):1041‐1047. doi:10.2967/jnumed.110.085100 21680680

[116] Kong G , Hofman MS , Murray WK , et al. Initial experience with gallium‐68 DOTA‐octreotate PET/CT and peptide receptor radionuclide therapy for pediatric patients with refractory metastatic neuroblastoma. J Pediatr Hematol Oncol. 2016;38(2):87‐96. doi:10.1097/MPH.0000000000000411 26296147

[117] Gains JE , Moroz V , Aldridge MD , et al. A phase IIa trial of molecular radiotherapy with 177‐lutetium DOTATATE in children with primary refractory or relapsed high‐risk neuroblastoma. Eur J Nucl Med Mol Imaging. 2020;47(10):2348‐2357. doi:10.1007/s00259-020-04741-x 32157433

[118] Pizzoferro M , Cassano B , Altini C , et al. Imaging post‐^177^Lu‐peptide receptor radionuclide therapy in a child with advanced progressive somatostatin‐receptor‐positive medulloblastoma. Eur J Nucl Med Mol Imaging. 2021;48(3):937‐939. doi:10.1007/s00259-020-04966-w 32767090

[119] Tsukamoto T , Miki Y . Imaging of pituitary tumors: an update with the 5th WHO classifications‐part 1. Pituitary neuroendocrine tumor (PitNET)/pituitary adenoma. Jpn J Radiol. 2023;41(8):789‐806. doi:10.1007/s11604-023-01400-7 36826759 PMC10366012

[120] Trouillas J , Jaffrain‐Rea ML , Vasiljevic A , Raverot G , Roncaroli F , Villa C . How to classify the pituitary neuroendocrine tumors (PitNET)s in 2020. Cancers Basel. 2020;12(2):514. doi:10.3390/cancers12020514 32098443 PMC7072139

[121] Cuevas‐Ramos D , Fleseriu M . Somatostatin receptor ligands and resistance to treatment in pituitary adenomas. J Mol Endocrinol. 2014;52(3):R223‐R240. doi:10.1530/JME-14-0011 24647046

[122] Behling F , Honegger J , Skardelly M , et al. High expression of somatostatin receptors 2A, 3, and 5 in corticotroph pituitary adenoma. Int J Endocrinol. 2018;2018:1763735. doi:10.1155/2018/1763735 30627156 PMC6304820

[123] Chinezu L , Vasiljevic A , Jouanneau E , et al. Expression of somatostatin receptors, SSTR2A and SSTR5, in 108 endocrine pituitary tumors using immunohistochemical detection with new specific monoclonal antibodies. Hum Pathol. 2014;45(1):71‐77. doi:10.1016/j.humpath.2013.08.007 24182563

[124] Hofland LJ , Lamberts SW . The pathophysiological consequences of somatostatin receptor internalization and resistance. Endocr Rev. 2003;24(1):28‐47. doi:10.1210/er.2000-0001 12588807

[125] Marques P . The effects of peptide receptor radionuclide therapy on the neoplastic and normal pituitary. Cancers Basel. 2023;15(10):2710. doi:10.3390/cancers15102710 37345047 PMC10216433

[126] Burman P , Casar‐Borota O , Perez‐Rivas LG , Dekkers OM . Aggressive pituitary tumors and pituitary carcinomas: from pathology to treatment. J Clin Endocrinol Metab. 2023;108(10):1585‐1601. doi:10.1210/clinem/dgad098 36856733 PMC10271233

[127] Lugowska I , Becker JC , Ascierto PA , et al. Merkel‐cell carcinoma: ESMO‐EURACAN clinical practice guideline for diagnosis, treatment and follow‐up. ESMO Open. 2024;9(5):102977. doi:10.1016/j.esmoop.2024.102977 38796285 PMC11145756

[128] Akaike T , Qazi J , Anderson A , et al. High somatostatin receptor expression and efficacy of somatostatin analogues in patients with metastatic Merkel cell carcinoma. Br J Dermatol. 2021;184(2):319‐327. doi:10.1111/bjd.19150 32320473 PMC7891857

[129] Askari E , Moghadam SZ , Wild D , et al. Peptide receptor radionuclide therapy in Merkel cell carcinoma: a comprehensive review. J Nucl Med Technol. 2023;51(1):22‐25. doi:10.2967/jnmt.122.264904 36195446

[130] Savelli G , Zaniboni A , Bertagna F , et al. Peptide receptor radionuclide therapy (PRRT) in a patient affected by metastatic breast cancer with neuroendocrine differentiation. Breast Care. 2012;7(5):408‐410. doi:10.1159/000343612 24647781 PMC3518936

[131] Yasukawa R , Kawamoto B , Muraoka K , Nakamura K , Honda M , Takenaka A . Excellent response to ^177^Lu‐DOTATATE peptide receptor radionuclide therapy in a patient with treatment‐related neuroendocrine prostate cancer with urinary retention and rectal obstruction: a case report. Yonago Acta Med. 2024;67(3):266‐269. doi:10.33160/yam.2024.08.010 39176189 PMC11335926

[132] Zhang J , Kulkarni HR , Singh A , Baum RP . Twelve‐year survival of a patient with lymph node, pulmonary, bone, cardiac and Intraspinal metastases of a rectal neuroendocrine neoplasm treated with peptide receptor radionuclide therapy‐the value of salvage peptide receptor radionuclide therapy. Clin Nucl Med. 2020;45(4):198‐200. doi:10.1097/RLU.0000000000002905 31876836

[133] Weller M , Albert NL , Galldiks N , et al. Targeted radionuclide therapy for gliomas: emerging clinical trial landscape. Neuro Oncol. 2024;26(suppl):S208‐S214. doi:10.1093/neuonc/noae125 39107236 PMC11631073

[134] Heute D , Kostron H , von Guggenberg E , et al. Response of recurrent high‐grade glioma to treatment with (90)Y‐DOTATOC. J Nucl Med. 2010;51(3):397‐400. doi:10.2967/jnumed.109.072819 20150267

[135] di Santo G , Santo G , Sviridenko A , Virgolini I . Peptide receptor radionuclide therapy combinations for neuroendocrine tumours in ongoing clinical trials: status 2023. Theranostics. 2024;14(3):940‐953. doi:10.7150/thno.91268 38250038 PMC10797289

[136] Oddstig J , Bernhardt P , Nilsson O , Ahlman H , Forssell‐Aronsson E . Radiation induces up‐regulation of somatostatin receptors 1, 2, and 5 in small cell lung cancer in vitro also at low absorbed doses. Cancer Biother Radiopharm. 2011;26(6):759‐765. doi:10.1089/cbr.2010.0921 22060188

[137] Pawlik TM , Keyomarsi K . Role of cell cycle in mediating sensitivity to radiotherapy. Int J Radiat Oncol Biol Phys. 2004;59(4):928‐942. doi:10.1016/j.ijrobp.2004.03.005 15234026

[138] Gong L , Zhang Y , Liu C , Zhang M , Han S . Application of Radiosensitizers in cancer radiotherapy. Int J Nanomedicine. 2021;16:1083‐1102. doi:10.2147/IJN.S290438 33603370 PMC7886779

[139] Yadav MP , Ballal S , Bal C . Concomitant ^177^Lu‐DOTATATE and capecitabine therapy in malignant paragangliomas. EJNMMI Res. 2019;9(1):13. doi:10.1186/s13550-019-0484-y 30725219 PMC6365580

[140] Cavalcanti E , Armentano R , Valentini AM , Chieppa M , Caruso ML . Role of PD‐L1 expression as a biomarker for GEP neuroendocrine neoplasm grading. Cell Death Dis. 2017;8(8):e3004. doi:10.1038/cddis.2017.401 PMC5596583 28837143

[141] Rösner E , Kaemmerer D , Neubauer E , Sänger J , Lupp A . Prognostic value of PD‐L1 expression in bronchopulmonary neuroendocrine tumours. Endocr Connect. 2021;10(2):180‐190. doi:10.1530/EC-20-0540 33475525 PMC7983515

[142] Kim C , Liu SV , Subramaniam DS , et al. Phase I study of the ^177^Lu‐DOTA^0^‐Tyr^3^‐Octreotate (lutathera) in combination with nivolumab in patients with neuroendocrine tumors of the lung. J Immunother Cancer. 2020;8(2):e000980. doi:10.1136/jitc-2020-000980 32616557 PMC7333915

[143] Ray Chaudhuri A , Nussenzweig A . The multifaceted roles of PARP1 in DNA repair and chromatin remodelling. Nat Rev Mol Cell Biol. 2017;18(10):610‐621. doi:10.1038/nrm.2017.53 28676700 PMC6591728

[144] Rauch H , Kitzberger C , Janghu K , et al. Combining [^177^Lu]Lu‐DOTA‐TOC PRRT with PARP inhibitors to enhance treatment efficacy in small cell lung cancer. Eur J Nucl Med Mol Imaging. 2024;51:4099‐4110. doi:10.1007/s00259-024-06844-1 39023784 PMC11527929

[145] Bushnell DL , Bodeker KL , O'Dorisio TM , et al. Addition of 131I‐MIBG to PRRT (90Y‐DOTATOC) for personalized treatment of selected patients with neuroendocrine tumors. J Nucl Med. 2021;62(9):1274‐1277. doi:10.2967/jnumed.120.254987 33517327 PMC8882893

[146] Goodhead DT , Thacker J , Cox R . Weiss lecture. Effects of radiations of different qualities on cells: molecular mechanisms of damage and repair. Int J Radiat Biol. 1993;63(5):543‐556. doi:10.1080/09553009314450721 8099101

[147] Goodhead DT . Mechanisms for the biological effectiveness of high‐LET radiations. J Radiat Res. 1999;40:1‐13. doi:10.1269/jrr.40.s1 10804988

[148] Kassis AI . Therapeutic radionuclides: biophysical and radiobiologic principles. Semin Nucl Med. 2008;38(5):358‐366. doi:10.1053/j.semnuclmed.2008.05.002 18662557 PMC2584872

[149] Narayanan PK , Goodwin EH , Lehnert BE . Alpha particles initiate biological production of superoxide anions and hydrogen peroxide in human cells. Cancer Res. 1997;57(18):3963‐3971. 9307280

[150] Navalkissoor S , Grossman A . Targeted alpha particle therapy for neuroendocrine tumours: the next generation of peptide receptor radionuclide therapy. Neuroendocrinology. 2019;108(3):256‐264. doi:10.1159/000494760 30352433

[151] Han G , Hwang E , Lin F , et al. RYZ101 (Ac‐225 DOTATATE) opportunity beyond gastroenteropancreatic neuroendocrine tumors: preclinical efficacy in small‐cell lung cancer. Mol Cancer Ther. 2023;22(12):1434‐1443. doi:10.1158/1535-7163.MCT-23-0029 37616528

[152] Tafreshi NK , Pandya DN , Tichacek CJ , et al. Preclinical evaluation of [^225^Ac]Ac‐DOTA‐TATE for treatment of lung neuroendocrine neoplasms. Eur J Nucl Med Mol Imaging. 2021;48(11):3408‐3421. doi:10.1007/s00259-021-05315-1 33772332 PMC12866175

[153] Demirci E , Alan Selçuk N , Beydağı G , et al. Initial findings on the use of [^225^Ac]Ac‐DOTATATE therapy as a theranostic application in patients with neuroendocrine tumors. Mol Imaging Radionucl Ther. 2023;32(3):226‐232. doi:10.4274/mirt.galenos.2023.38258 37870290 PMC10600558

[154] Yadav MP , Ballal S , Sahoo RK , Bal C . Efficacy and safety of ^225^Ac‐DOTATATE targeted alpha therapy in metastatic paragangliomas: a pilot study. Eur J Nucl Med Mol Imaging. 2022;49(5):1595‐1606. doi:10.1007/s00259-021-05632-5 34837103 PMC8626283

[155] Kunikowska J , Królicki L . Targeted α‐emitter therapy of neuroendocrine tumors. Semin Nucl Med. 2020;50(2):171‐176. doi:10.1053/j.semnuclmed.2019.11.003 32172802

[156] Gape PMD , Schultz MK , Stasiuk GJ , Terry SYA . Towards effective targeted alpha therapy for neuroendocrine tumours: a review. Pharmaceuticals Basel. 2024;17(3):334. doi:10.3390/ph17030334 38543120 PMC10974115

[157] Reubi JC , Maecke HR . Approaches to multireceptor targeting: hybrid radioligands, radioligand cocktails, and sequential radioligand applications. J Nucl Med. 2017;58(suppl. 2):10‐16. doi:10.2967/jnumed.116.186882 28864606

[158] Rottenburger C , Nicolas GP , McDougall L , et al. Cholecystokinin 2 receptor agonist ^177^Lu‐PP‐F11N for radionuclide therapy of medullary thyroid carcinoma: results of the Lumed phase 0a study. J Nucl Med. 2020;61(4):520‐526. doi:10.2967/jnumed.119.233031 31519804 PMC7198370

[159] Eigler C , McDougall L , Bauman A , et al. Radiolabeled somatostatin receptor antagonist versus agonist for peptide receptor radionuclide therapy in patients with therapy‐resistant meningioma: Promenade phase 0 study. J Nucl Med. 2024;65(4):573‐579. doi:10.2967/jnumed.123.266817 38423782

